# Integrating Remote Sensing and Machine Learning to Project Global Habitat Suitability and Productivity of Chinese Fir Under Climate Change

**DOI:** 10.1002/ece3.73757

**Published:** 2026-06-04

**Authors:** Jiejie Sun, Xiao He, Tongli Wang, Qian Wang, Boran Liu, Jing Qian, Dawei Luo, Hui Xia, Xuan Xu, Xiangdong Lei, Jiaen Zhang, Weifeng Wang, Ming Xu

**Affiliations:** ^1^ Guangdong‐Hong Kong Joint Laboratory for Carbon Neutrality Jiangmen Laboratory of Carbon Science and Technology Jiangmen Guangdong Province China; ^2^ Co‐Innovation Center for Sustainable Forestry in Southern China, College of Biology and the Environment Nanjing Forestry University Nanjing Jiangsu China; ^3^ Department of Forest and Conservation Sciences, Faculty of Forestry & Environmental Stewardship University of British Columbia Vancouver British Columbia Canada; ^4^ Key Laboratory of Forest Management and Growth Modelling, State Forestry and Grassland Administration, Institute of Forest Resource Information Techniques Chinese Academy of Forestry Beijing China; ^5^ Department of Ecology, College of Natural Resources and Environment South China Agricultural University Guangzhou Guangdong China

**Keywords:** Chinese fir, climate change, *Cunninghamia lanceolata*, ecological niche modeling, net primary productivity

## Abstract

Chinese fir ( *Cunninghamia lanceolata* ) is China's most widely planted industrial plantation species, yet productivity declines have been reported in several regions. Climate change is likely to intensify these risks by simultaneously reshaping climatic suitability and limiting sustainable net primary productivity (NPP), but their combined effects have not been quantified in a global, multi‐model framework. Here, we integrate ecological niche models (ENMs) with multiple machine‐learning models for NPP, calibrated using 3139 occurrence records, MODIS‐derived NPP, and 37 climate–soil covariates. Future projections are driven by an ensemble of 13 CMIP6 GCMs under SSP245 and SSP585. Across scenarios, suitable habitat is projected to contract in the current core region of southern China while expanding poleward, with new suitability in North China, the eastern United States, and South America. By 2081–2100, habitat losses account for 16%–18% of the current suitable area, partly offset by gains in newly suitable regions equivalent to 35%–45% of the current suitable area. Within today's planting footprint, total NPP is projected to decline by 6%–12% (≈1.3–5.6 × 10^9^ t·year^−1^) relative to the current total NPP under the same footprint. In contrast, tracking future suitable zones under an idealized assisted‐migration scenario could potentially increase total NPP by 15%–20% relative to the current total NPP. Warm‐season precipitation and temperature‐regime variability (annual range and isothermality) emerge as dominant controls, highlighting coupled hydrothermal constraints. This integrated assessment provides strategic evidence for prioritizing climate‐forward plantation siting.

## Graphical abstract

We integrated ecological niche modeling (MaxEnt) with a remote sensing–driven machine‐learning productivity model (BRT), using 3139 Chinese fir occurrences, MODIS‐derived NPP, and 37 climate–soil predictors to project future suitability and productivity under a 13‐GCM CMIP6 ensemble (SSP245/SSP585). Projections indicate contraction in the current core region of southern China and poleward expansion, with total NPP declining by ~6%–12% if plantations remain in today's footprint but increasing by ~15%–20% if planting zones track future suitability (assisted migration). Warm‐season precipitation and temperature‐regime variability (temperature annual range and isothermality) emerge as dominant controls, providing a transferable basis for climate‐smart plantation zoning and conservation‐aware management.

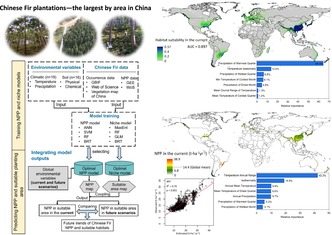

## 1 Introduction

Plantation forests hold only 7% of global forest areas but provide nearly 40% of the world's timber supply (FAO 2020). Due to ecological and environmental considerations, many countries have begun to prohibit the cutting of natural forests (Zhang and Chen 2021; King 2023). Thus, forest plantations will play a more important role in the future global timber supply. However, in the context of rapid global climate change, the habitat suitability and productivity of plantation forests face enormous challenges (Dai et al. 2016; Zhang et al. 2022).

To assess climate‐change impacts on plantation forests, two approaches are commonly used, but both have limitations. One approach relies on long‐term plot‐based ground investigations (Liu et al. 2018; Wang et al. 2021) or flux tower observations (Campioli et al. 2016; Wen et al. 2014); however, these observations cover only a small proportion of forest area and therefore cannot by themselves support forest plantation productivity projections at the global scale. Another approach uses terrestrial biosphere models to simulate future yields at regional scales (Volk et al. 2018). However, this approach is constrained by regional limitations in plant physiological parameters (Morin et al. 2007; Valade et al. 2014), and model uncertainty remains high (Kang et al. 2017; Zhao et al. 2009). In addition, previous productivity‐related studies often neglected climate‐driven habitat change (Cook‐Patton et al. 2020; Ploton et al. 2020), which may increase uncertainty. Although a recent study suggested that changes in habitat suitability largely determine future plantation productivity (Sun et al. 2021), that approach did not directly account for climate‐driven changes in NPP.

As an important plantation forest species, Chinese fir ( *Cunninghamia lanceolata* ) is one of the most important plantation species in China and has been introduced to multiple regions worldwide, serving as a key model species for assessing climate‐change impacts on timber production (FAO 2020). Previous studies on Chinese fir have primarily examined stand‐management effects on productivity, including planting density (Liu et al. 2019; Yang et al. 2022) and thinning (Qu et al. 2022; Zhou, Cai, He, Wang, Wu, and Ma 2016), or projected future productivity at regional scales using ecosystem process‐based models (Lu et al. 2015). However, global‐scale projections that jointly account for climate‐driven habitat change and per‐area NPP remain limited. This creates a focused knowledge gap: it remains unclear whether future areas that are climatically suitable for Chinese fir will also sustain high productivity, or whether climate change will create spatial mismatches between suitability and productivity.

Here, we test the hypothesis that climate change will alter both the spatial distribution of climatically suitable habitat and per‐area NPP for Chinese fir, potentially generating mismatches between future suitability and productivity. We therefore develop a comprehensive assessment of climate‐change impacts on the climatic suitability and productivity of Chinese fir plantations at the global scale by addressing three questions: (1) What is the distribution pattern of NPP and suitable habitat of Chinese fir under current climate conditions? (2) What are the separate and joint impacts of climate change on the climatic suitability and NPP of Chinese fir plantations at the global scale? (3) To what extent could assisted migration to future climatically suitable areas potentially help offset projected productivity losses?

## 2 Materials and Methods

### 2.1 Data Collection

#### 2.1.1 Plantation Distribution and Productivity Data

**FIGURE 1:**
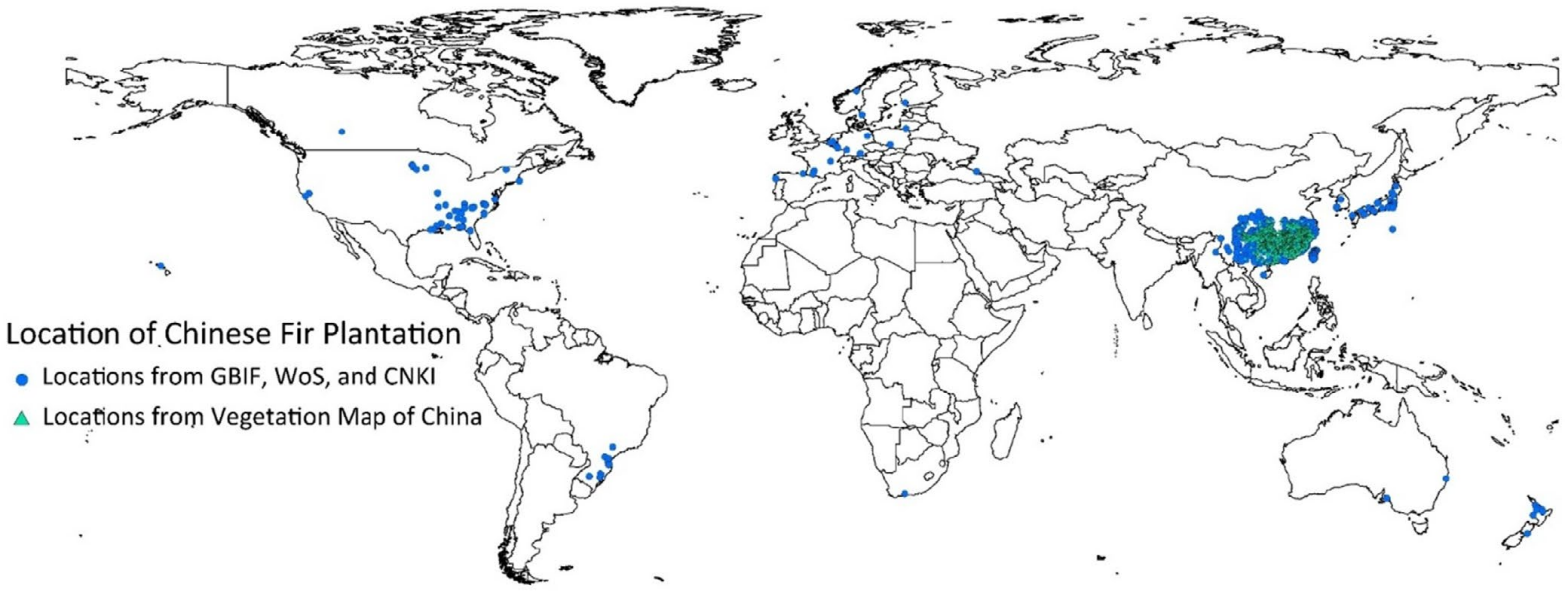
Locations of existing Chinese fir plantation worldwide.

Chinese fir originated in China and has been introduced to Vietnam, Japan, South Korea, Europe, North America, South America, and Australia in recent years (Figure 1). To develop ecological niche models (ENMs) to assess the climatic suitability and machine learning regression models to predict NPP for Chinese fir globally, we collected and compiled the *C. lanceolata* occurrence data from the Global Biodiversity Information Facility (GBIF, https://www.gbif.org), Web of Science (WoS; http://apps.webofknowledge.com/), and China National Knowledge Infrastructure (CNKI, https://www.cnki.net/). We also extracted Chinese fir distribution points from Vegetation Map of China followed Lu et al. (2015). To avoid sample duplication and overrepresentation, we used the “spThin” package in R to keep only one data point in each 2.5 arc minutes geographic grid (Aiello‐Lammens et al. 2015). After screening, 3139 locations (199 locations from GBIF, 2906 locations from Vegetation Map of China, 34 sites from WoS and CNKI) of *C. lanceolata* were used for model building. We inputted all the locations data into Google Earth Engine (GEE) to extract the NPP value for each point from the Moderate‐resolution Imaging Spectroradiometer (MODIS)‐based product named “MODIS/Terra Net Primary Production Gap‐Filled Yearly L4 Global 500” in GEE (Running and Zhao 2021). In this study, NPP is reported as dry‐matter biomass rather than carbon mass, with per‐area values expressed in t ha^−1^ year^−1^ and aggregated totals in t year^−1^. Given the uncertainty in a single year of remote sensing data, we extracted the mean value of MODIS NPP 2002–2020 for our modeling.

#### 2.1.2 Environmental Data

We obtained current and future climate data from WorldClim—Global Climate Data (https://www.worldclim.org/) (Fick and Hijmans 2017). Since CMIP6 data (Coupled Model Intercomparison Project Phase 6) offers more robust projections than CMIP5 due to better representations of physical processes and climate feedbacks (Su et al. 2021), we selected future climate data from CMIP6's general circulation models (GCMs). We downloaded data of 13 GCMs (each providing 19 climate variables) from WorldClim (Eyring et al. 2016; Fick and Hijmans 2017) and averaged them to construct an ensemble climate dataset, thereby reducing the uncertainty inherent in any single GCM (Table S1). The shared socioeconomic pathways (SSPs) of the CMIP6 make future scenarios more reasonable than the representative concentration pathway (RCP) of the CMIP5 (Su et al. 2021). Thus, we selected climate data predicted under SSP245 and SSP585 future climate change scenarios, representing the lowest and the highest impact of rising greenhouse gas concentrations, respectively. These climate data include average monthly climate data, such as minimum, mean, and maximum temperature and precipitation (Voldoire et al. 2019). The spatial resolution of all climatic variables used was 2.5 arc minutes.

The current climate data is the average for the years 1970–2000. We selected future climate data from 2021–2040, 2041–2060, 2061–2080, and 2081–2100 for our study. In total, 21 climatic variables were chosen for our model input (Table S2). The spatial resolution of all climatic variables used was 2.5 arc minutes. In addition to 19 climatic factors from WorldClim (Fick and Hijmans 2017), we also calculated annual mean vapor pressure deficit (VPD) and vapor pressure deficit of warmest quarter (VPDWQ). We calculated the annual/warmest quarter mean saturated vapor pressure (SVP) according to the temperature‐SVP empirical model (Lowe 1977).

The Harmonized World Soil Database (HWSD, http://www.fao.org/land‐water/databases‐and‐software/hwsd/en/) is one of the most comprehensive soil databases. We treated soil variables as constant and used them in our current and future modeling. Fifteen soil variables from the HWSD contained a data layer of key soil physicochemical properties at a spatial resolution of 30 arc seconds. We obtained the global groundwater percentile (GWP) dataset from the NASA GRACE project (Li et al. 2019). This dataset covers the period from February 3, 2003 to May 2021 and provides GWP data at a 7‐day interval (https://nasagrace.unl.edu/globaldata/). To facilitate our analysis, we first converted the GWP data into annual values for each year. Subsequently, we incorporated these annual values into our modeling efforts. All predictor layers were resampled/aligned to a common 2.5 arc‐minute grid before modeling, and grid cells with missing predictor values were excluded from model fitting and projection.

### 2.2 Ecological Niche Modeling

**FIGURE 2:**
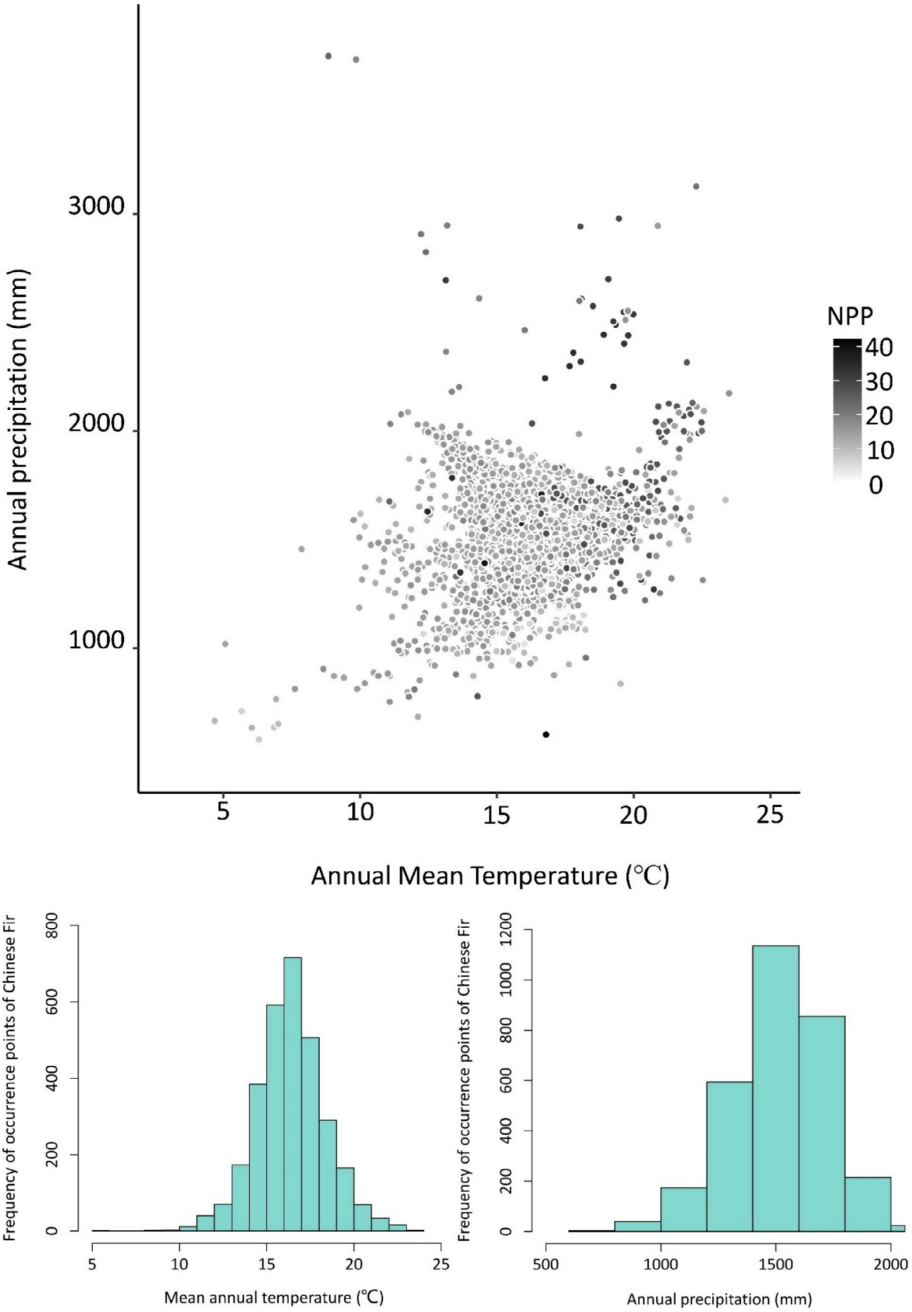
Distribution patterns of Chinese fir plantation forest locations in mean annual temperature (MAT) and annual precipitation (AP).

**FIGURE 3:**
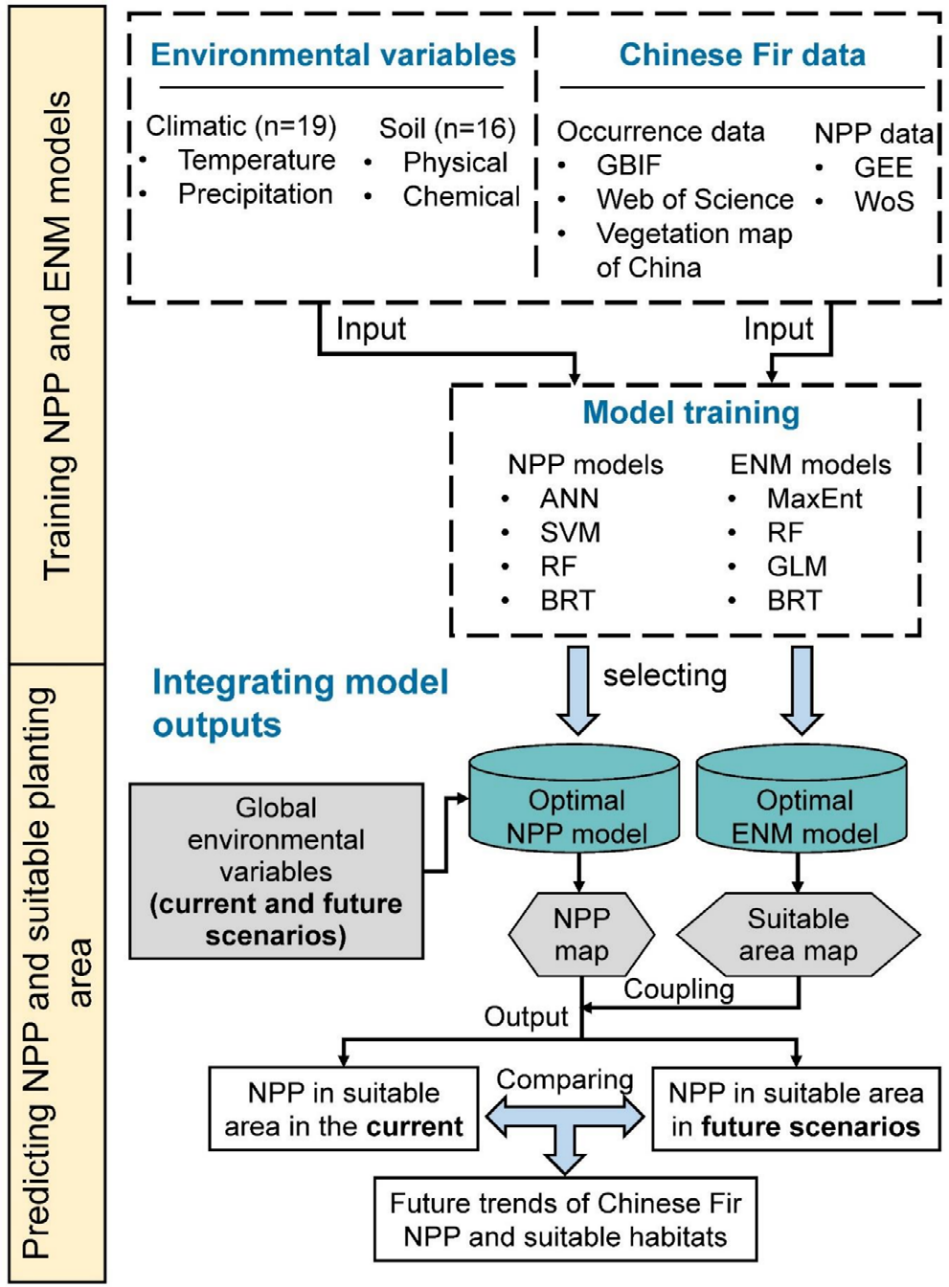
Framework of the procedure for modeling the net primary productivity (NPP) and suitable area for Chinese fir plantations at the global scale.

To examine the impact of climate change on the climatic suitability of Chinese fir, we developed ecological niche models (ENMs). Before model construction, we first evaluated the reliability of the occurrence data. Although Chinese fir has been introduced by humans for more than 2000 years, its current distribution range has not exceeded its possible historical natural distribution range (Li 2015). The existing Chinese fir occurrence points showed a normal distribution on the mean annual temperature (MAT) and annual precipitation (AP) gradient to some extent (Figure 2), indicating that our data set is relatively reasonable and reliable. Therefore, we consider it reasonable to use the historical occurrence records of Chinese fir as input for our ecological niche modeling. As outlined in Figure 3, we calibrated ecological niche models to estimate climatic suitability under current and future climates.

To simplify the analysis and reduce the risk of overfitting caused by multicollinearity, we first built a correlation matrix with all environmental variables for each occurrence point and excluded one variable from each pair of variables that were highly correlated to each other (Pearson correlation coefficient > 0.85) (Zhang et al. 2022). Preference was given to directly measured climate and annual variables over the derived and monthly variables. Permutation importance was used to evaluate the predictive contribution of each environmental variable. To further reduce the number of environmental variables, we discarded variables with little contribution to the testing model. Finally, we retained 10 environmental variables for the development of the models for performance comparison. We first compared several ENMs using a low‐collinearity set of 10 predictors; after selecting MaxEnt as the best performer, we expanded the candidate pool and applied contribution‐ and correlation‐based screening, yielding 13 predictors for the final MaxEnt model.

These selected environmental variables were used as predictors to calibrate four widely applied ENM algorithms: the Maximum Entropy model (MaxEnt), random forest (RF), generalized linear models (GLM), and boosted regression trees (BRT). Before modeling, we randomly selected 1500 points as the pseudo‐absence background point. For each model, we selected 75% of the data points as the training set and 25% of the data points as the validation set and repeated the model simulation five times for cross‐validation. Model performance was evaluated based on three widely accepted metrics: the Area Under the Receiver Operating Characteristic Curve (AUC), the True Skill Statistic (TSS), and the Kappa coefficient (Phillips and Dudik 2008; Karger et al. 2021). The mean values of these metrics across the five replicates were calculated for each model. The best‐performing model was identified by jointly considering all three indices to ensure both discriminative power, accuracy, and agreement consistency.

**FIGURE 4:**
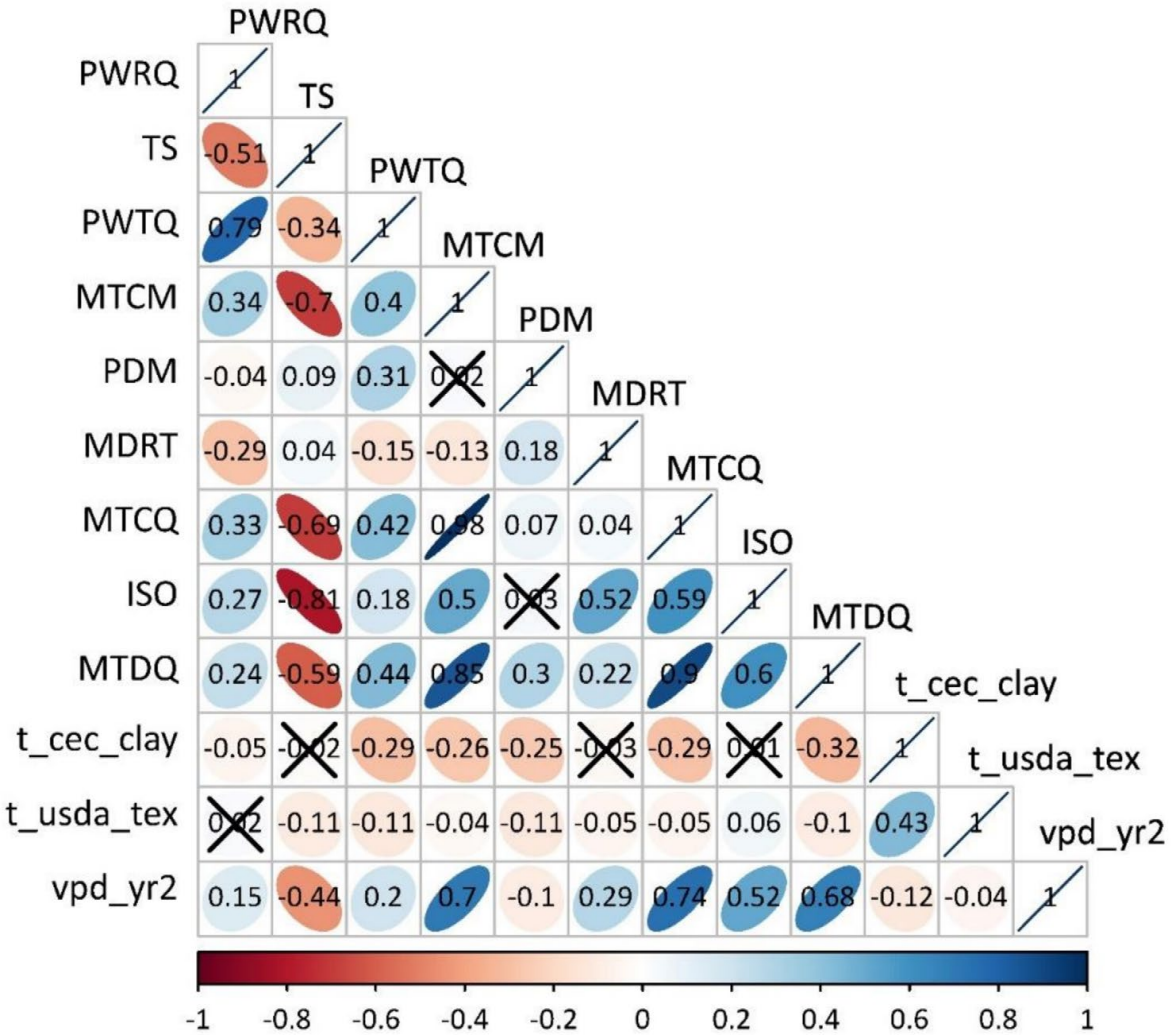
The Pearson correlation coefficients of selected environmental variables for ENMs.

After comparing the performance of multiple ENM algorithms (Table 1), we selected the MaxEnt model as the optimal model. Therefore, we will focus on the steps we took to establish the MaxEnt model. We implemented MaxEnt (v3.4.1) via R 4.5.0 (RStudio 2025.05.0) using the dismo, raster, and rJava packages to model the current and future climate suitability of global Chinese fir plantations, as it is one of the most robust ENMs (Padalia et al. 2014; Hernandez et al. 2006) and has been widely used to test the climatic suitability of forests under global climate change (Mori et al. 2021; Gomes et al. 2019; Jones et al. 2022; Betts et al. 2022). MaxEnt uses 75% of the total sample points for training and the remaining 25% for testing. The model was built with 21 climate and 15 soil variables for the current period. Climate variables with no contribution to the models were discarded after three iterations. Then, we analyzed the Pearson Correlations Coefficient between the remaining climate variables and removed one of the paired variables with a correlation coefficient above 0.85 (Figure 4). Finally, we selected nine climate variables, two soil variables, and annual mean VPD (Table S2, with #) to build the final model. The output value of this model was between 0 and 1. The larger the value of a particular geographic grid, the higher the environmental suitability for this species. We defined a grid with a suitability value equal or greater than 0.1 as a “suitable grid”, and a grid with a value smaller than 0.1 as a “non‐suitable grid” following a previous study (Sun et al. 2021). A simple sensitivity analysis showed that the proportion of occurrence points retained within the predicted suitable area was highly similar under thresholds of 0.10 and 0.17 (Table S3); given that Chinese fir is a managed plantation species, we therefore retained 0.10 as a slightly more inclusive operational threshold. To visualize spatial shifts in the climatically permissible range, we provide gridded maps of binary suitable areas for each future scenario and time slice (Figure S2), obtained by thresholding continuous suitability at 0.1 as justified above.

**TABLE 1:** Predictive performance of four ecological niche model algorithms. Values of the area under the ROC curve (AUC), true skill statistics (TSS), and Cohen's kappa are the means from five independent repeated runs of each model; larger values indicate stronger discriminatory power and classification agreement.

Model	AUC	TSS	Kappa
MaxEnt	0.897	0.704	0.595
Random forest	0.844	0.602	0.653
Generalized linear model	0.813	0.437	0.546
Boosted regression tree	0.807	0.576	0.633

### 2.3 Productivity Modeling

**FIGURE 5:**
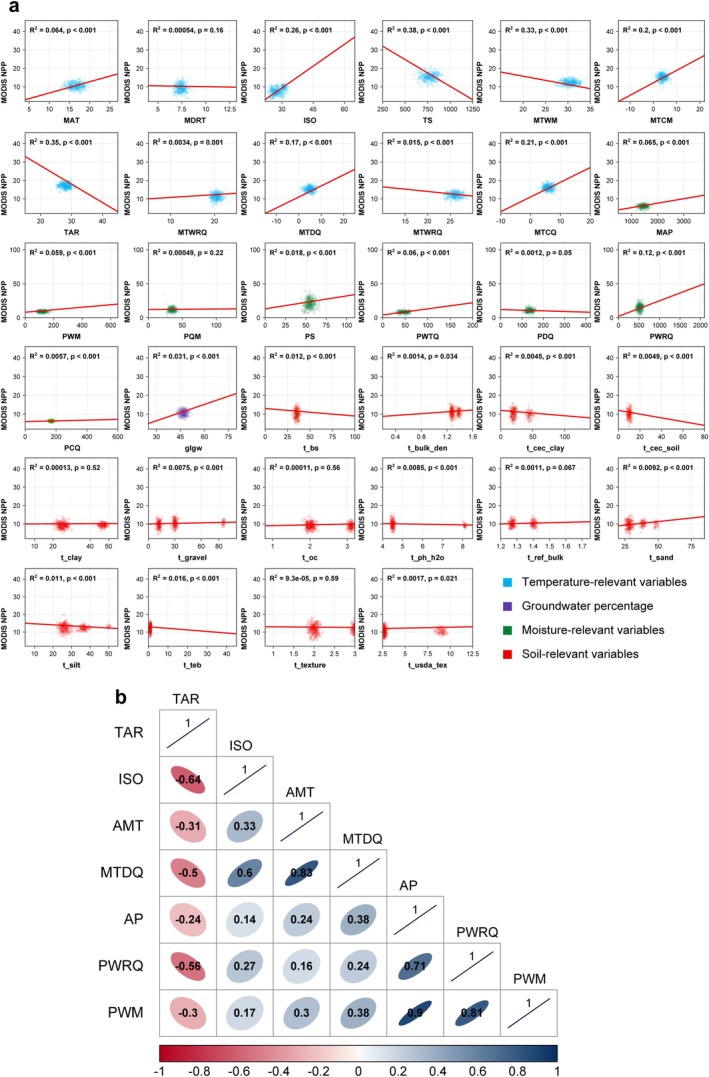
The relationships between MODIS NPP of Chinese fir plantation forests and environmental variables (a). The Pearson Correlation Coefficients of selected environmental variables for NPP regression model (b).

We considered four commonly used machine learning (ML) algorithms, including Artificial Neural Network (ANN), Support Vector Machine (SVM), Random Forest (RF), and Boosted Regression Tree (BRT), to model the relations between the NPP of Chinese fir plantations and environmental variables (e.g., climate, and soil). During the model building, we first selected the independent variables according to the following rules: (1) the absolute value of the Pearson correlation coefficient between each independent variable and the NPP was greater than 0.2 (Figure 5); (2) eliminated independent variables with a variance inflation factor (VIF) > 10 (Chen et al. 2018). We finally selected seven variables (Table S2, with *) for building the final models. The correlations between these variables were all smaller than 0.8 (Figure 5). To characterize environmental controls and how they may shift, we summarize the top‐ranked predictors of the NPP model for the current climate and show end‐of‐century anomalies of those predictors under SSP245 and SSP585 (Figure S5).

**FIGURE 6:**
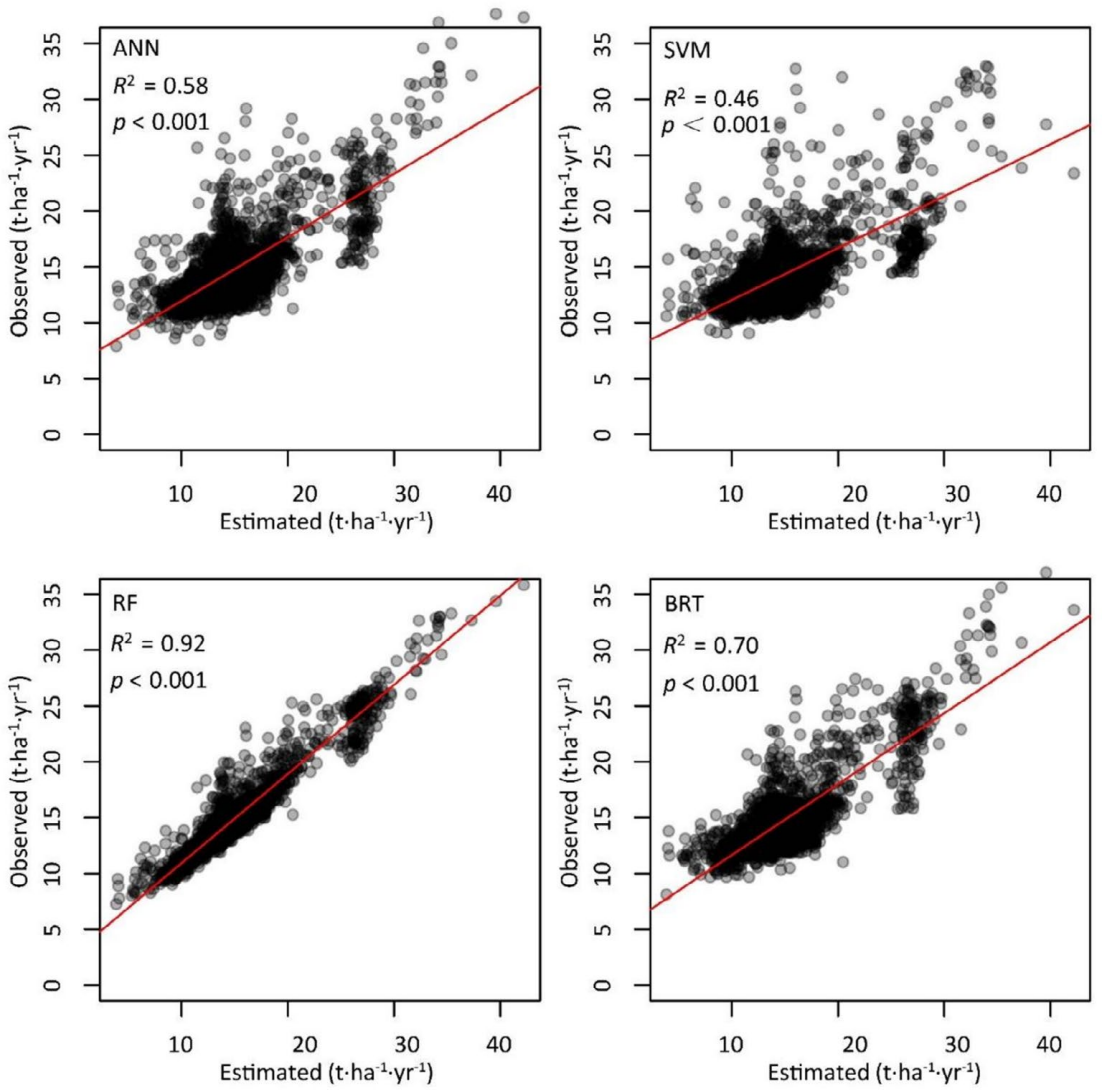
Observed versus estimated NPP of Chinese fir plantations under current conditions for four machine‐learning models: Artificial Neural Network (ANN), Support Vector Machine (SVM), Random Forest (RF), and Boosted Regression Tree (BRT). Observed values were derived from MODIS NPP.

We compared the *R* ^2^ of the full‐data modeling and the *R* ^2^ of 10‐fold cross‐validation (Figure 6, Tables S4 and S5) and selected the optimal model and chose the Boosted Regression Tree (BRT) algorithm to build our final model for projections of the global NPP of Chinese fir under future scenarios globally. The details and hyperparameters for the model establishment are described in the Supporting Information (Table S6).

We used the following equations to evaluate the performance of the final productivity model including *R* ^2^ (1), root mean square error (RMSE (2)), relative root mean square error (rRMSE (3)), and mean absolute error (MAE (4)):

(1) $$R^{2}=1-\frac{\sum {\left({\hat{y}}_{i}-y_{i}\right)}^{2}}{\sum {\left(y_{i}-\bar{y}\right)}^{2}}$$

(2) $$\text{RMSE}=\sqrt{\frac{\sum {\left({\hat{y}}_{i}-y_{i}\right)}^{2}}{n}}$$

(3) $$\text{rRMSE}=\frac{\text{RMSE}}{\bar{y}}\times 100$$

(4) $$\mathrm{MAE}=\frac{\sum \left|{\hat{y}}_{i}-y_{i}\right|}{n}$$

where $y_{i}$ is the NPP training value of the *i*‐th pixel; ${\hat{y}}_{i}$ is the predicted NPP value of the *i*‐th pixel; $\bar{y}$ is the average value of predicted NPP of all the pixels; *n* is the number of pixels.

### 2.4 Future Productivity Projections

**FIGURE 7:**
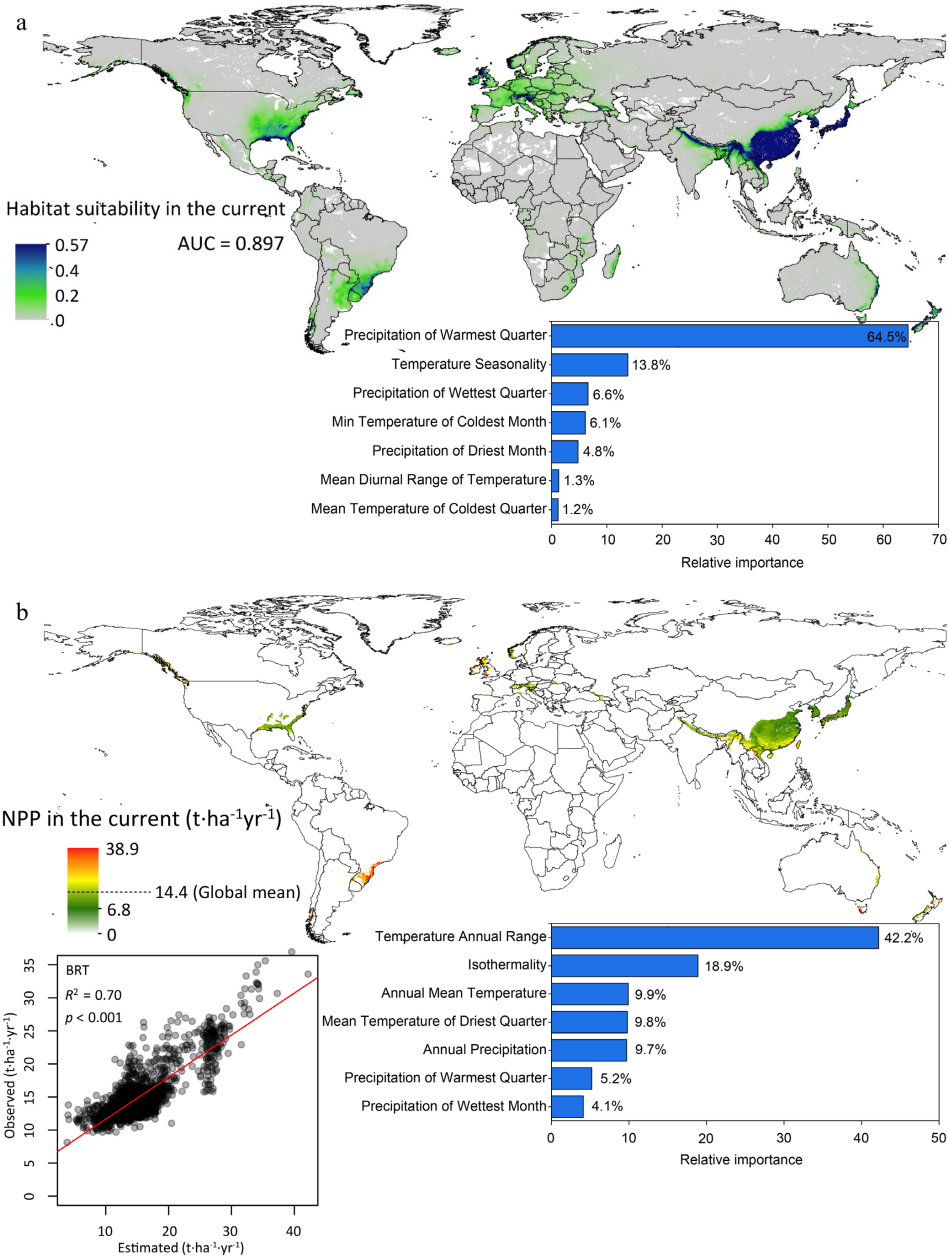
Predicted habitat suitability (a) and predicted net primary productivity (NPP) (b) of Chinese fir plantations under the current climate condition and their driving environmental factors with relative importance.

**FIGURE 8:**
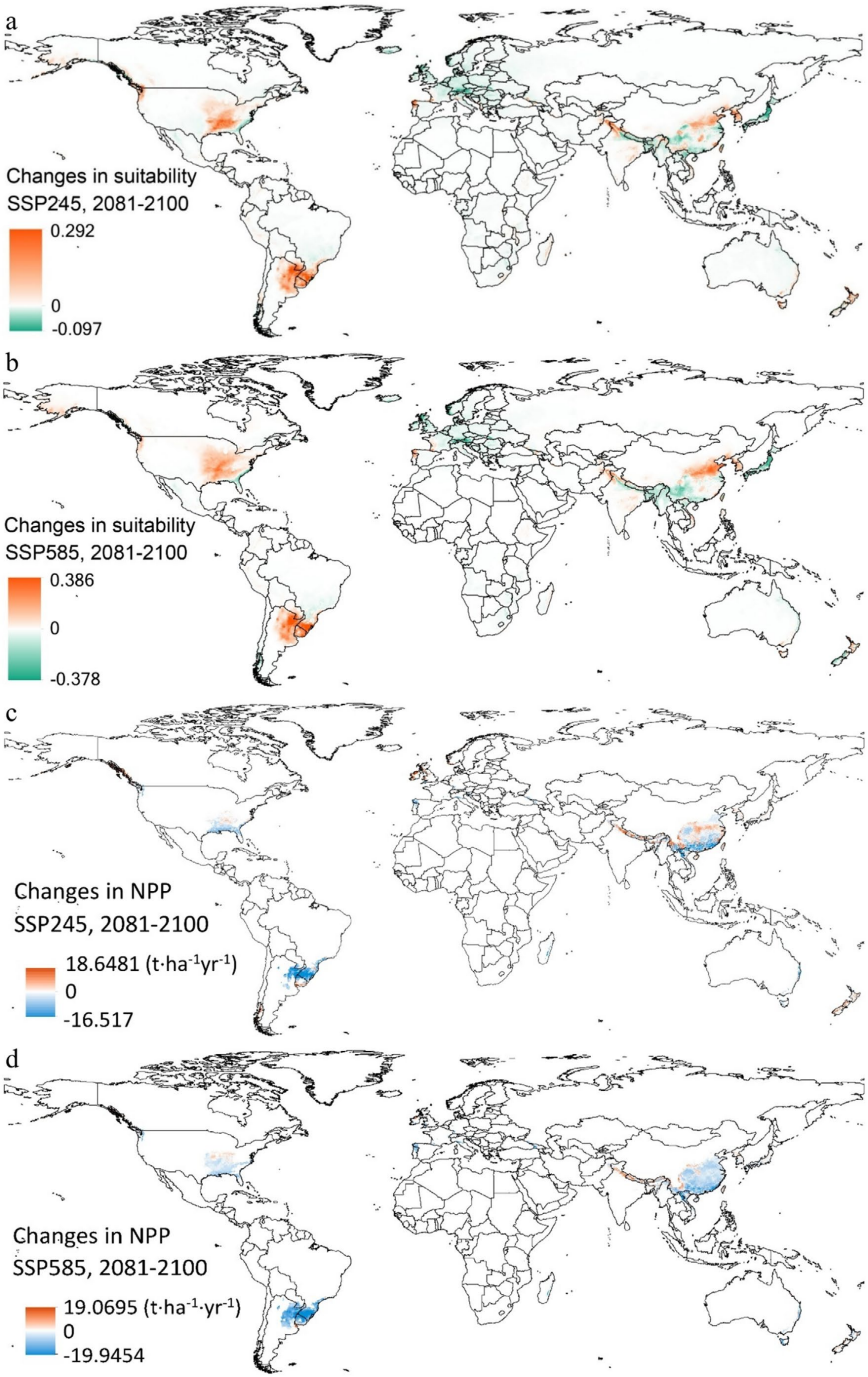
Projected changes in habitat suitability of Chinese fir plantations (a and b) and NPP (c and d) under the scenarios of SSP245 (a and c) and SSP585 (b and d).

We used the BRT model to project the future total NPP of Chinese fir plantations globally. We evaluated two deployment scenarios. S0 maintained the current planting footprint, with per‐area NPP projected under future climate conditions. S1 allowed planting areas to shift according to projected future climatic suitability while also allowing per‐area NPP to change. For each scenario, we summed NPP across the corresponding scenario‐specific grid cells for the current and future periods. In Figures 7b and 8c,d, we display NPP maps obtained by masking projected NPP with the habitat‐suitability maps (i.e., intersecting NPP with areas deemed climatically suitable). To provide additional information to readers, we also map the unconstrained NPP projection, that is, predictions prior to intersecting with habitat suitability, for the present and each future scenario (Figure S1). These maps reveal geographic productivity gradients independent of niche limits. To illustrate realized production under future climates, we also display maps of projected NPP intersected with areas classified as suitable (threshold 0.1) for each scenario and time slice (Figure S3). Because S1 is an exploratory upper‐bound scenario, establishment feasibility, invasion risk, land availability, and policy constraints were considered beyond the scope of the present modeling framework.

## 3 Results

### 3.1 Suitable Habitat and Productivity Under Current Climate Condition

The AUC of our ecological niche model was 0.897, indicating a high model accuracy. The habitat suitability (probability of occurrence) of Chinese fir ranged from 0 to 0.57 globally (Figure 7a). The global distribution pattern of habitat suitability had a high overlap with the occurrence points (Figures 1 and 7a), confirming that our model well captured the habitat suitability. The environmental variables with major contributions to the model included the precipitation of warmest quarter (PWRQ) (64.5%), temperature seasonality (13.8%), precipitation of wettest quarter (6.6%), and min temperature of coldest month (6.1%) (Figure 7a).

The Boosted Regression Tree (BRT) algorithm showed a strong model fit (*R* ^2^ = 0.70, 10‐CV *R* ^2^ = 0.52 ± 0.057, Tables S4 and S5, Figure 6). The projected high‐NPP areas (NPP > 14.4 t·ha^−1^·year^−1^) of Chinese fir plantations mainly distributed in southern China, South America, and Europe (Figure 7b). The top important environmental variables included temperature annual range (42.2%), Isothermality (18.9%), and annual mean temperature (9.9%).

### 3.2 Projected Changes in Suitable Areas and Productivity in the Future

Our projections indicate that the habitat suitability of Chinese fir in central and southwestern China (the main production region) is projected to decline by 2081–2100 under both SSP245 and SSP585 scenarios (Figure 8a,b). Similar decreases are evident in the nearer‐term periods (Figure S2). These losses suggest that approximately one‐sixth of the current suitable area may become climatically unsuitable under future climate conditions. The models also project a shift of suitable habitat for Chinese fir from lower to higher latitudes under both SSP245 and SSP585 scenarios, with a stronger trend under SSP585. The central and eastern regions of the United States and the southern parts of South America are projected to contain areas with higher predicted climatic suitability for this species.

The NPP of Chinese fir in the main production area in China is projected to decrease in the future, with a stronger decline under SSP585 than under SSP245 (Figure 8c,d). For completeness, we also provide realized‐NPP maps (projected NPP masked by suitability) for each of the four future time slices (Figure S3). Specifically, the projected NPP of Chinese fir plantations in much of southern China, the southern part of the east coast of the United States, and southern South America is projected to decline by 0–19.9 t·ha^−1^·year^−1^. Increases in predicted NPP are projected in specific regions, including the middle and lower reaches of the Yangtze River in China, the east coast of the United States, and southwest China. Moisture and thermal‐variability proxies (e.g., TAR, ISO, and MAT) emerge among the highest‐importance predictors of current NPP, and their projected anomalies by 2100 track the spatial pattern of NPP change (Figure S5).

### 3.3 Potential Strategies to Mitigate the Impact of Climate Change

**FIGURE 9:**
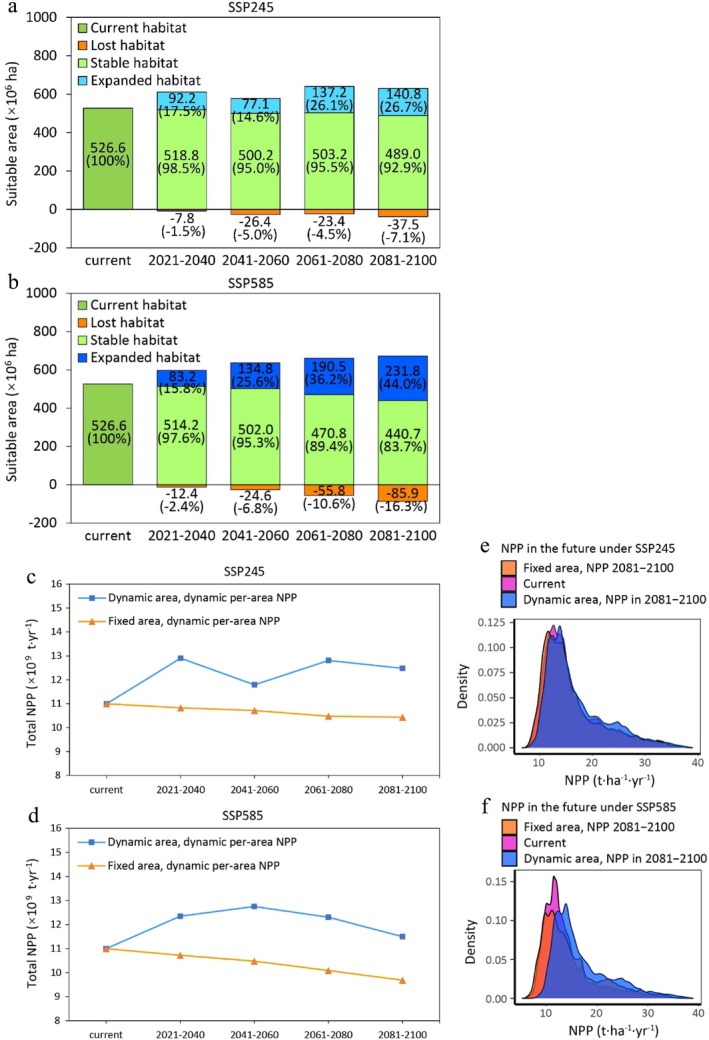
Projected changes in planting area and total NPP of Chinese fir under two management scenarios. S0 maintains the current planting footprint and allows future NPP to change within that footprint. S1 allows planting areas to shift according to projected future climatic suitability. Panels (a, b) show changes in suitable area, (c, d) total NPP, and (e, f) NPP‐density distributions in 2100 under SSP245 and SSP585. Percentage changes in area and total NPP are expressed relative to the current baseline.

Under the SSP585, 2081–2100, the model projected an expansion of 231.8 × 10^6^ ha of suitable habitat (an increase of 44%) and a loss of 85.9 × 10^6^ ha (16.3%) suitable habitat (Figure 9a,b); a similar trend (relatively weaker) is shown under SSP245. Although the projected expansion was larger than the loss of the suitable habitat in the future, expansion of suitable habitats does not mean that Chinese fir will automatically disperse to the expanded suitable habitats within decades, but the loss of suitable habitats implies elevated risk of growth decline and mortality, especially under compound heat–drought stress.

The total NPP of Chinese fir was projected to decrease by about 6%–12% (1.3–5.6 × 10^9^ t·year^−1^) by 2081–2100 under S0, in which the current planting footprint is maintained while per‐area NPP changes under future climate. Under S1, in which planting areas are allowed to shift according to projected future climatic suitability, total NPP was projected to increase by about 15%–20% by 2081–2100 (blue line in Figure 9c,d). Raster cells retained under S1 have a higher frequency (density) of high‐NPP values (Figure 9e,f), which helps explain the projected increase in total NPP. The projected total NPP under the two planting scenarios showed different trends, indicating that assisted migration guided by projected future climate‐suitable habitats could potentially help offset part of the negative impact of climate change on Chinese fir productivity.

## 4 Discussion

This study provides an integrated assessment of climate risks to Chinese fir industrial forest plantation production by jointly modeling climatic suitability and per‐area productivity. We compiled global occurrence records, satellite‐constrained NPP, and gridded climate and soil covariates, trained an ecological niche model (ENM) to map suitable area and a boosted regression tree (BRT) model to predict NPP, and projected both under CMIP6 SSP245 and SSP585. We then evaluated two deployment scenarios: one maintaining the current planting footprint (S0) and the other allowing planting areas to shift according to projected future climatic suitability (S1). This joint framing yields a consistent, decision‐relevant signal across scenarios: S0 is projected to reduce end‐century total NPP by ≈6%–12%, whereas S1, as an idealized upper‐bound scenario, could potentially increase total NPP by ≈15%–20%. Warm‐season precipitation, temperature annual range, and isothermality emerge as leading drivers, identifying potential levers for climate‐forward siting and water‐balance management. Taken together, this work highlights strategic options that require local validation before operational implementation.

### 4.1 Climate Risks to Production Emerge From the Joint Dynamics of Suitability and Per‐Area NPP

Our analysis integrates habitat suitability and per‐area productivity to evaluate climate risks to Chinese fir plantations at the global scale. Two robust signals emerge across scenarios and time slices (Figures 7, 8, 9). First, if planting remains within today's footprint, total production declines toward the end of the century as large portions of the current core belt in southern China experience reduced suitability and NPP. Second, if planting tracks projected suitability—i.e., assisted migration to climatically favorable areas—aggregate production could potentially be maintained or even increased because future high‐NPP clusters become more spatially concentrated. Together, these results move beyond single‐axis assessments—either “area only” or “yield only”—and show that the production trajectory of plantation forests is co‐determined by range shifts and physiological constraints.

**FIGURE 10:**
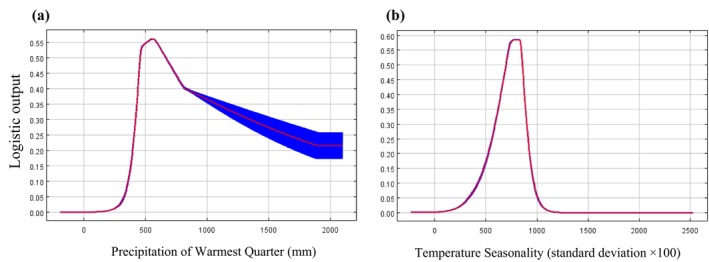
Response curves of the existence probability (habitat suitability) of Chinese fir to major environmental factors under current climatic conditions.

Mechanistically, our models recover driver–response patterns that align with plantation physiology and site water balance. On the suitability axis, our ENM identifies precipitation of the warmest quarter (PWRQ) as the dominant control (Figure 7a), consistent with the view that hot summers constrain Chinese fir growth when moisture becomes limiting (Wang et al. 2022; O'Donnel and Ignizio 2012). Suitability peaks at intermediate PWRQ (≈500–1000 mm; Figure 10), a range that matches the species' extensive planting on mountainous terrain (Lu et al. 2015), where soils are often shallower than in adjacent lowlands (Patton et al. 2018). Under consecutive sunny days in June–August, shallow soils deplete quickly, and near‐surface vapor pressure deficit rises (Lu et al. 2015), together producing episodic drought stress. Because multiple CMIP6 models project declining warm‐season precipitation across parts of southern China (Figure S4), our maps show corresponding losses of suitable habitat in that region toward century's end.

On the productivity axis, temperature annual range (TAR) and isothermality (ISO) emerge as leading predictors of per‐area NPP (Figure 7b; Figure S5). Chinese fir attains higher NPP where interannual temperature variability is smaller and ISO is higher, indicating a tighter coupling between diurnal and annual thermal regimes. ISO is defined as 100 × (mean diurnal range ÷ annual temperature range); larger values imply relatively stable year‐to‐year temperatures (Fick and Hijmans 2017). The positive association between ISO and NPP likely reflects thermally benign conditions that reduce respiration losses at night and support daytime carbon gain within favorable temperature windows (Zhang et al. 2023). Spatial patterns of end‐century anomalies in these drivers co‐locate with projected NPP changes (Figure 8c,d; Figure S5), reinforcing that warm‐season moisture limitation and thermal variability are first‐order bottlenecks for Chinese fir growth under warming.

### 4.2 Chinese Fir Management Implications: Where and How to Adapt

Our projections do not prescribe a single pathway, but they point to near‐term levers for adapting Chinese fir plantations. First, prioritize climate‐forward site selection: assisted movement of seed sources and planting toward zones with more favorable hydrothermal conditions may help recapture productivity losses seen in the current core belt (see Figure 7a,b; Figures S4 and S5). This aligns with operational guidance for forest‐assisted migration and climate‐based seed transfer that favors modest, risk‐aware shifts in provenance and planting zones rather than large jumps (Palik et al. 2022; Stanturf et al. 2024). Within China, targeting landscapes with reliable warm‐season moisture (or irrigation potential) and moderated thermal variability is consistent with observed climate sensitivities of Chinese fir growth (Wang et al. 2022). Because suitability dips where warm‐season water is limiting, silvicultural water‐balance tools are pivotal: retaining surface residues/organic matter and maintaining some understory cover can cool and humidify the sub‐canopy and reduce evaporative demand (Greiser et al. 2024). Where feasible, micro‐/drip‐irrigation during establishment can bridge dry spells—an approach already tested for tree planting and restoration in northern China and highlighted in recent water‐saving planning (Zhang et al. 2025).

Stand structure and genetics should be tuned to local thermal and moisture regimes. In warmer, drier margins, avoiding overly dense spacing and using earlier, lighter thinning reduces competition for soil water and has repeatedly been shown to improve drought resistance and recovery at stand level; conversely, slightly higher densities can be sustained where moisture is reliable and thermal variability is muted (Sohn et al. 2016; Sankey and Tatum 2022). On the genetic side, deploying provenances or improved material screened for heat/episodic‐drought tolerance on advancing fronts, while maintaining within‐site genetic diversity, hedges risk by preserving adaptive potential and reducing pest/disease vulnerability (Gao et al. 2021; Kremer et al. 2025). Finally, use driver‐based monitoring for adaptive control: managers can track proximal hydroclimatic proxies—especially warm‐season VPD and standardized VPD indices—to trigger operational responses (e.g., thinning timing, irrigation triggers, seedling protection) before growth losses accumulate (Corak et al. 2025; Greiser et al. 2024; Grossiord et al. 2020).

### 4.3 Land‐Use and Biodiversity Safeguards When Expanding Into New Zones

Land‐use change is a leading driver of biodiversity loss; any expansion guided by our projections should therefore be paired with explicit safeguards (Jaureguiberry et al. 2022; Maestre et al. 2025). Our maps indicate several regions where future suitability rises, but the biodiversity context differs and should shape siting and management. In lower‐conflict landscapes, especially parts of northern China where large areas are already converted and where abandonment of cropland has been mapped at scale, prioritizing already‐converted or low‐conservation‐value lands can minimize additional biodiversity impacts while supplying biomass; where feasible, target marginal or abandoned fields, shelterbelt mosaics, or other degraded lands rather than intact habitats (Tu et al. 2024; Ye et al. 2024). In high‐value regions—for example, parts of Uruguay and the eastern United States that emerge as potentially suitable—adopt strict siting standards: avoid recognized hotspots and intact habitats, use site‐level screening tools, and favor restoration‐compatible configurations (set‐asides, buffers, landscape corridors) on already converted lands. For the U.S., NatureServe's Map of Biodiversity Importance provides high‐resolution guidance on areas critical for at‐risk species in the East; for Uruguay, recent syntheses and datasets underscore the conservation significance and threats to native grasslands, strengthening the case for caution (Grattarola et al. 2020; Ramírez and Säumel 2022). Projected newly suitable pixels identified under S1 should not be interpreted as universally available or advisable for plantation establishment, because urban land, cropland, protected areas, and other competing land uses were not explicitly masked in the present analysis. We emphasize that S1 represents an idealized upper‐bound scenario rather than an immediately achievable management pathway; implementation would depend on seed availability, provenance selection, establishment time lags, land‐use constraints, and institutional feasibility (Palik et al. 2022; Wotherspoon et al. 2025).

At the site scale, silviculture can reduce local biodiversity costs. Frequent shrub removal and broad‐scale weeding tend to depress understory diversity and simplify structure; where ecological sensitivity is higher, retain or restore native understory patches, stagger weeding to maintain heterogeneity, and incorporate set‐aside strips/microhabitats (Zhou, Cai, He, Wang, Wu, and Ma 2016). Mixed‐species or mosaic designs that include Chinese fir with locally adapted companions improve structural and compositional diversity and can enhance water‐use efficiency and climate resilience; converting pure stands to multi‐layered mixed stands is increasingly supported by manipulative studies and meta‐analyses (Zhang et al. 2024; Xiong et al. 2024). For Chinese fir plantations, retain harvest residues after felling and avoid blanket understory removal; where soils are degraded, add organic amendments/biochar at site preparation to rebuild soil C and water‐holding and curb fertilizer inputs (Yang et al. 2024; Xiong et al. 2024).

While debates persist on plantation–biodiversity trade‐offs, a growing body of work shows that careful siting and diversification can narrow these trade‐offs, allowing planted forests to provide substantial biomass and local economic benefits without undermining high‐value ecosystems (Hua et al. 2022; Xi et al. 2022).

### 4.4 Brief Caveats and Future Work

Our predictions are designed for strategic planning rather than fine‐scale prescriptions. ENM outputs represent potential climatic suitability rather than realized plantations. In China and Vietnam, projected suitability broadly aligns with the current planting footprint, whereas suitability emerging outside the current range (e.g., the eastern United States, Europe, and South America) should be interpreted as climatic analogs and evaluated case‐by‐case given local soils, biotic pressures, management feasibility, and regulatory/biodiversity constraints. Although occurrence records were spatially thinned, they remain regionally clustered, and the use of random cross‐validation may therefore overestimate ENM performance because nearby training and test points can share similar environmental conditions; spatially explicit validation would provide a stricter assessment in future work. Several factors not fully encoded could modulate realized outcomes at local scales: (i) developmental stage/stand age and rotation effects can alter climate sensitivities across ontogeny; (ii) inter‐ and intra‐specific interactions (e.g., competition with companion species in mixed stands; pest–host dynamics) were not mechanistically modeled; (iii) management heterogeneity (fertility programs, thinning, irrigation) is not fully represented by our remote‐sensing‐constrained NPP; because the productivity model was trained and evaluated against MODIS‐derived NPP, the projected NPP should be interpreted mainly as relative spatial patterns rather than precise absolute yields, and uncertainties related to MODIS NPP, stand age, management, disturbance, and land‐cover classification may also introduce bias into the projections; (iv) soil properties were treated as static layers, so long‐term feedbacks from repeated rotations are not captured; however, because only a small number of soil predictors were retained in the final model, this simplification is unlikely to strongly affect the broad‐scale patterns of our projections, although it may still introduce local biases, especially under the assisted migration scenario; and (v) while the CMIP6‐GCM ensemble reduces dependence on any single climate model and helps smooth idiosyncratic errors, it may also mask inter‐model disagreement, so scenario and model spread still persists, especially for hydroclimate. These limitations point to clear next steps: integrate age‐structured yield functions and management covariates; combine SDMs with process‐based modules for moisture stress and pests; incorporate dynamic soil constraints; and validate projections with coordinated plot networks and flux towers along climate gradients.

## 5 Conclusions

This study integrates 3139 Chinese fir occurrence records, MODIS‐derived NPP, and 37 climate and soil covariates to project climatic suitability and productivity under CMIP6 SSP245 and SSP585 using ENM and BRT models. By jointly assessing suitability and per‐area NPP, we show that future suitable habitat is projected to shift poleward, whereas total NPP within the current planting footprint is projected to decline by ≈6%–12% by 2081–2100; under the idealized assisted‐migration scenario, tracking projected suitability could potentially increase total NPP by ≈15%–20%. Warm‐season precipitation, temperature annual range, and isothermality emerged as key drivers, indicating coupled moisture and thermal constraints on future productivity. These results provide a strategic basis for climate‐forward site selection, water‐balance management, and climate‐smart genetic deployment, while emphasizing that assisted migration and expansion into newly suitable regions require local validation, land‐use assessment, and biodiversity safeguards.

## Author Contributions

**Jiejie Sun:** conceptualization (lead), data curation (lead), formal analysis (lead), funding acquisition (equal), investigation (lead), methodology (lead), project administration (equal), software (lead), visualization (lead), writing – original draft (lead), writing – review and editing (lead). **Xiao He:** data curation (equal), methodology (equal), software (equal), visualization (equal), writing – review and editing (equal). **Tongli Wang:** data curation (equal), resources (equal), writing – review and editing (equal). **Qian Wang:** visualization (equal). **Boran Liu:** data curation (equal). **Jing Qian:** writing – review and editing (equal). **Dawei Luo:** writing – review and editing (equal). **Hui Xia:** software (equal), visualization (equal). **Xuan Xu:** writing – review and editing (equal). **Xiangdong Lei:** writing – review and editing (equal). **Jiaen Zhang:** writing – review and editing (equal). **Weifeng Wang:** funding acquisition (equal), resources (equal), supervision (equal), writing – review and editing (equal). **Ming Xu:** funding acquisition (equal), supervision (equal), writing – review and editing (equal).

## Funding

This work was supported by National Natural Science Foundation of China (32502051), Guangdong‐Hong Kong‐Macao joint Laboratory (2023B1212120003), Guangdong Talent Program (2023JC10N060), Guangdong Science and Technology Program (2022B1212040001), Special Fund for Science and Technology Innovation Strategy of Guangdong Province (2022660500250009604), China Postdoctoral Science Foundation (2024M750957), Guangdong Provincial Postdoctoral International Training Program, and National Key Research, and Development Program of China (2021YFD220040402).

## Conflicts of Interest

The authors declare no conflicts of interest.

## Supporting information


**Table S1:** 13 general circulation models (GCMs) of Coupled Model Intercomparison Project Phase 6 (CMIP6) from the WorldClim.
**Table S2:** Candidate environmental variables for machine‐learning NPP models and ENMs in this study. Final variables used in the machine‐learning NPP models are marked with (*), and final variables used in the ENMs are marked with (#).
**Table S3:** Sensitivity analysis of alternative habitat‐suitability thresholds. Number and percentage of Chinese fir occurrence points falling within the predicted suitable area under two data‐driven logistic thresholds and the threshold adopted in this study.
**Table S4:** Results of full‐data regression model of Artificial neural network (ANN), Support vector machine (SVM), Random Forest (RF), Boosted Regression Trees (BRT).
**Table S5:** Results of 10‐fold cross‐validation regression model of Artificial neural network (ANN), Support vector machine (SVM), Random Forest (RF), Boosted Regression Trees (BRT).
**Table S6:** Hyper‐parameters of four ML regression model of Artificial neural network (ANN), Support vector machine (SVM), Random Forest (RF), Boosted Regression Trees (BRT).
**Figure S1:**. The global projected NPP map of Chinese fir plantation forests in the current and the future scenarios (not masked by suitable habitats).
**Figure S2:**. The global suitable areas of Chinese fir in the future scenarios.
**Figure S3:**. The global projected NPP of Chinese fir masked by suitable areas in the future scenarios.
**Figure S4:**. Major environmental factors of ENM of Chinese fir plantation forests in the current and their changes in the future scenario of SSP245 and SSP585 under 2100.
**Figure S5:**. Major environmental factors of NPP regression model for Chinese fir plantation forests in the current and their changes in the future scenario of SSP245 and SSP585 under 2100.

## Data Availability

Our compiled occurrence and environment data for our modeling are available from the open data repository (https://figshare.com/s/a62ddc7f0254d3d5870f). The species occurrence data for Chinese fir originally from Global Biodiversity Information Facility (GBIF, https://www.gbif.org), Web of Science (http://apps.webofknowledge.com/), and China National Knowledge Infrastructure (CNKI, https://www.cnki.net/). The climate data is originally from WorldClim version 2.1 (https://worldclim.org/data/index.html). Soil data is originally from the Harmonized World Soil Database (HWSD, http://www.fao.org/land‐water/databases‐and‐software/hwsd/en/).
