# Supplementary material for: Integrating Remote Sensing and Machine Learning to Project Global Habitat Suitability and Productivity of Chinese Fir Under Climate Change
Source: Ecol Evol. 2026 Jun 4;16(6):e73757. doi: 10.1002/ece3.73757 (PMC13290655; doi:10.1002/ece3.73757)
Supplement: Supplementary file 1 — Table S1: 13 general circulation models (GCMs) of Coupled Model Intercomparison Project Phase 6 (CMIP6) from the WorldClim. Table S2: Candidate environmental variables for machine‐learning NPP models and ENMs in this study. Final variables used in the machine‐learning NPP models are marked with (*), and final variables used in the ENMs are marked with (#). Table S3: Sensitivity analysis of alternative habitat‐suitability thresholds. Number and percentage of Chinese fir occurrence points falling within the predicted suitable area under two data‐driven logistic thresholds and the threshold adopted in this study. Table S4: Results of full‐data regression model of Artificial neural network (ANN), Support vector machine (SVM), Random Forest (RF), Boosted Regression Trees (BRT). Table S5: Results of 10‐fold cross‐validation regression model of Artificial neural network (ANN), Support vector machine (SVM), Random Forest (RF), Boosted Regression Trees (BRT). Table S6: Hyper‐parameters of four ML regression model of Artificial neural network (ANN), Support vector machine (SVM), Random Forest (RF), Boosted Regression Trees (BRT). Figure S1:. The global projected NPP map of Chinese fir plantation forests in the current and the future scenarios (not masked by suitable habitats). Figure S2:. The global suitable areas of Chinese fir in the future scenarios. Figure S3:. The global projected NPP of Chinese fir masked by suitable areas in the future scenarios. Figure S4:. Major environmental factors of ENM of Chinese fir plantation forests in the current and their changes in the future scenario of SSP245 and SSP585 under 2100. Figure S5:. Major environmental factors of NPP regression model for Chinese fir plantation forests in the current and their changes in the future scenario of SSP245 and SSP585 under 2100. [file ECE3-16-e73757-s001.docx]

**Supplementary information for manuscript titled ‘Integrating remote sensing and machine learning to project global habitat suitability and productivity of Chinese fir under climate change’**

Jiejie Sun^1, 2, 4^, Xiao He^3^, Tongli Wang^2^, Qian Wang^1^, Boran Liu^1^, Jing Qian^2^, Dawei Luo^2^, Hui Xia^1^, Xuan Xu^1^, Xiangdong Lei^3^, Jiaen Zhang^5^, Weifeng Wang^1, *^, Ming Xu^1, *^

^1^ Guangdong-Hong Kong Joint Laboratory for Carbon Neutrality, Jiangmen Laboratory of Carbon Science and Technology, Jiangmen 529199, Guangdong Province, China

^2^ Co-Innovation Center for Sustainable Forestry in Southern China, College of Biology and the Environment, Nanjing Forestry University, Nanjing, Jiangsu 210037, China

^3^ Department of Forest and Conservation Sciences, Faculty of Forestry, University of British Columbia, Vancouver, BC V6T 1Z4, Canada

^4^ State Key Laboratory of Efficient Production of Forest Resources, Key Laboratory of Forest Management and Growth Modelling, State Forestry and Grassland Administration, Institute of Forest Resource Information Techniques, Chinese Academy of Forestry, Beijing 100091, China

^5^ Department of Ecology, College of Natural Resources and Environment, South China Agricultural University, Guangzhou, Guangdong 510642, China

**^*^Corresponding authors:** Weifeng Wang, Nanjing Forestry University, 159 Longpan Road, Xuanwu, Nanjing, China (Phone: +86 25-85428015, +86 15805152400; Email: [wang.weifeng@njfu.edu.cn](mailto:wang.weifeng@njfu.edu.cn)); Ming Xu, Jiangmen Laboratory of Carbon Science and Technology, Hong Kong University of Science and Technology (Guangzhou), 29 Jinzhou Road, Jiangmen, Guangdong, China (Phone: +86 13552593273; Email: [mingxu@igsnrr.ac.cn](mailto:mingxu@igsnrr.ac.cn)).

Jiejie Sun and Xiao He contributed equally to this work.

**Table S1.** 13 general circulation models (GCMs) of Coupled Model Intercomparison Project Phase 6 (CMIP6) from the WorldClim.

| GCM | Description |
| --- | --- |
| ACCESS-CM2 | Australian CMIP6 atmosphere–ocean coupled model developed by CSIRO and BoM. |
| BCC-CSM2-MR | Chinese medium‑resolution CMIP6 climate system model from Beijing Climate Center. |
| CMCC-ESM2 | Italian Earth system model v2 with interactive carbon and aerosol cycles. |
| EC-Earth3-Veg | European consortium model coupling atmosphere, ocean and dynamic vegetation modules. |
| FIO-ESM-2-0 | Chinese ocean‑centric Earth system model version 2 for CMIP6 analyses. |
| GISS-E2-1-G | NASA GISS climate model E2.1‑G using HYCOM ocean component. |
| HadGEM3-GC31-LL | UK Met Office HadGEM3 GC3.1 low‑resolution global coupled model. |
| INM-CM5-0 | Russian INM climate model 5.0 with updated sea‑ice and aerosols. |
| IPSL-CM6A-LR | French IPSL CM6A low‑resolution Earth system model with interactive aerosols. |
| MIROC6 | Japanese MIROC6 coupled model integrating atmosphere, ocean and land processes. |
| MPI-ESM1-2-HR | German MPI high‑resolution Earth system model 1.2 for CMIP6 HighResMIP. |
| MRI-ESM2-0 | Japanese MRI Earth system model 2.0 improving ENSO and QBO simulation. |
| UKESM1-0-LL | UK Earth system model 1.0 low‑resolution with full carbon–climate feedbacks. |

**Table S2.** Candidate environmental variables for machine-learning NPP models and ENMs in this study. Final variables used in the machine-learning NPP models are marked with (*), and final variables used in the ENMs are marked with (#).

| Abbreviation | Variables Description | Units | Abbreviation | Variables Description | Units |
| --- | --- | --- | --- | --- | --- |
| MAT* | Mean Annual Temperature | ℃ | MTCQ# | Mean Temperature of Coldest Quarter | ℃ |
| MDRT# | Mean Diurnal Range of Temperature | ℃ | AP* | Annual Precipitation | mm |
| ISO*# | Isothermality (MDR/TAR) | / | PWM* | Precipitation of Wettest Month | mm |
| TS# | Temperature Seasonality | / | PDM# | Precipitation of Driest Month | mm |
| MTWM | Max Temperature of Warmest Month | ℃ | PS | Precipitation Seasonality | mm |
| MTCM# | Min Temperature of Coldest Month | ℃ | PWTQ# | Precipitation of Wettest Quarter | mm |
| TAR* | Temperature Annual Range (MTWM-MTCM) | ℃ | PDQ | Precipitation of Driest Quarter | mm |
| MTWTQ | Mean Temperature of Wettest Quarter | ℃ | PWRQ*# | Precipitation of Warmest Quarter | mm |
| MTDQ*# | Mean Temperature of Driest Quarter | ℃ | PCQ | Precipitation of Coldest Quarter | mm |
| MTWRQ | Mean Temperature of Warmest Quarter | ℃ | AMVPD# | Annual Mean Vapor Pressure Deficit | hPa |
| VPDWM | Vapor Pressure Deficit of Warmest Month | hPa | T-CEC-CLAY# | Topsoil cation exchange capacity of (clay) | cmol/kg |
| T-GRAVEL | Topsoil Gravel Content | % | T-CEC-SOIL | Topsoil cation exchange capacity (soil) | cmol/kg |
| T-SAND | Topsoil Sand Fraction | % | T-BS | Topsoil Base Saturation | % |
| T-SILT | Topsoil Silt Fraction | % | T-TEB | Topsoil TEB | % |
| T-CLAY | Topsoil Clay Fraction | % | T-CaCO_3_ | Topsoil Calcium Carbonate | % |
| T_BULK_DENSITY | Topsoil Bulk Density | % | T-CASO_4_ | Topsoil Gypsum | % |
| T-OC | Topsoil Organic Carbon | % | T-ESP | Topsoil Sodicity (ESP) | % |
| T-PH-H_2_O | Topsoil pH (H_2_O) | -log(H^+^) | T-ECE | Topsoil Salinity (Elco) | dS/m |
| T_USDA_TEX_CLASS# | Topsoil USDA Texture Classification | / |  |  |  |

**Table S3.** Sensitivity analysis of alternative habitat-suitability thresholds. Number and percentage of Chinese fir occurrence points falling within the predicted suitable area under two data-driven logistic thresholds and the threshold adopted in this study.

| Description | Logistic threshold | Points in suitable area (%) |
| --- | --- | --- |
| Maximum training sensitivity plus specificity | 0.17 | 3243 (97.7) |
| Maximum test sensitivity plus specificity | 0.10 | 3270 (98.6) |

**NPP regression model development method**

The Artificial Neural Network (ANN), Support Vector Machine (SVM), Random Forest (RF), and Boosted Regression Tree (BRT) algorithms from machine learning (ML) models were chosen as it allows for non-linear, non-monotonic relationships between the target property and multiple covariates.

**ANN model:** ANN consists of the input, hidden and an output layer. The input layer is a matrix of *n×p* composed of independent variables, the hidden layer is composed of neural nodes, and the output layer is prediction results. After the independent variables matrix was input layer, each neural node in the hidden layer is obtained by linear transform and combine through the parameters named weight and bias. Finally, the output layer is obtained by the activation function. ANN can more efficiently approximate any nonlinear relationship (1). We used “nnet” package in R to implement our modeling and prediction (2). In order to eliminate the dimensional influence, all variables are standardized before modeling. For the ANN, there are two hyper-parameters to be optimized, namely, the weight decay rate (decay) and the number of hidden layer nodes (size). According to experience, set the decay from 0.001 to 1 with a step of 0.01; The size is set from 3 to 30 with a step of 1, and the activation function is *Sigmoid*. So, a total of 28,000 ANN models were built, we selected the ANN model with the best performance for NPP prediction comparison.

**SVM model:** SVM is a method to find the optimal hyper-plane that minimizes the total error of all samples out of the plane. However, in order to reduce the risk of over-fitting, *ε*-insensitive loss function that the error is not included when the absolute deviation between the observed and the predicted value is not greater than the *ε* that was given in advance is adopted (3). At the same time, SVR can use kernel method to find the linear relationship between independent and dependent variables in high-dimensional space to solve nonlinear problems. We used “e1071” package in R to implement our modeling and prediction (4). In order to eliminate the dimensional influence, all variables are standardized before modeling. For SVM, there are three hyper-parameters to be optimized, namely kernel function (kernel), kernel function coefficient (gamma) and allowable error (cost). According to experience, the kernel was set to radial or sigmoid; set gamma from 0.1 to 10 with a step of 0.1; set the cost from 0.1 to 10 with a step of 0.1. Therefore, a total of 20,000 SVR models were built, we selected the SVM model with the best performance for NPP prediction comparison.

**RF model:** RF is a machine learning algorithm that combines regression trees and bootstrap resampling (5), which can be used for classification and regression problems. The logic of the random forest algorithm to achieve regression are: 1) bootstrap sampling on the original training set (*n*×*p*), and get k training sets (*n*×*p*); 2) build a regression tree model for *k* training sets separately, thus obtaining *k* regression prediction results; 3) take the average of *k* regression results as the final prediction result (6). RF obtains high-accuracy prediction results by integrating multiple regression trees and improves the interpretability of the model by measuring the relative importance of independent variables (6). We used “randomForest” package in R to implement our modeling and prediction (7). For RF, the hyper-parameter (mtry) which is the number of independent variables randomly sampled by the decision tree during each split needs to be optimized, and the value is 1 to the number of independent variables. Therefore, a total of 6 RF models were built, we selected the RF model with the best performance for NPP prediction comparison.

**BRT model:** BRT improves the stability and accuracy by integrating the classification and regression tree (CART) and boosting algorithms (8). Different from RF, the learning of CART in BRT model is carried out sequentially (8). BRT gets a series of new training data sets by resampling the training data sets, and generates a corresponding regression tree according to each new training data set. The second regression tree is constructed according to the first regression tree residual error, and so on until the number of iterations specified by the user preset value (3). The final prediction result is the weighted average of all regression trees, in which the weight of each regression tree is contributed from its performance. We used “gbm” package in R to implement our modeling and prediction (9). For BRT, there are three hyper parameters that need to be optimized, namely, the interaction depth of variables (interaction.depth), the number of decision trees (n.trees), and the learning rate (shrinkage). According to experience, set the interaction.depth from 1 to 10, with a step of 1; set the n.trees from 50 to 500 with a step of 10; the shrinkage is set to 5 values, such as 0.05, 0.1, 0.01, 0.001 and 0.0001. Therefore, a total of 2300 BRT models were built, we selected the BRT model with the best performance for NPP prediction comparison.

We used current climate and soil factors (independent variables) and NPP (dependent variables) to establish the trained productivity machine learning models. Then, we used the model to predict the per area NPP of each grid under future climate conditions.

**Model selection**

In general, the RF and BRT algorithms are better than ANN and SVM in terms of R^2^, RMSE, rRMSE, and MAE. Among them, the R^2^ (0.90) of the full data modeling of the RF algorithm is higher than that of the BRT algorithm (0.70) (Table S4 and S5). The predicted and observed values of the RF and BRT algorithms are in good consistency (Figure 6 in manuscript). However, in the case of 10-fold cross-validation, the R^2^ of RF dropped significantly (reduce to 0.524), while the R^2^ of the BRT algorithm dropped relatively little (reduce to 0.522), that is, the R^2^ of the RF algorithm dropped by nearly 0.3, while the BRT algorithm dropped by nearly 0.17 (Table S4 and S5).

Therefore, this study believes that the NPP model of the BRT algorithm is more robust. This study ultimately selected the BRT algorithm to model and predict the global projected NPP of Chinese fir, and to project the model results to future climate conditions.

**Table S4.** Results of full-data regression model of Artificial neural network (ANN), Support vector machine (SVM), Random Forest (RF), Boosted Regression Trees (BRT).

| Model | Model accuracy with Full data | | | |
| --- | --- | --- | --- | --- |
|  | R^2^ | RMSE  (t·ha^-1^·yr^-1^) | rRMSE  (%) | MAE  (t·ha^-1^·a^-1^) |
| ANN | 0.58 | 2.66 | 18.32 | 1.90 |
| SVM | 0.45 | 3.04 | 20.95 | 2.01 |
| RF | 0.90 | 1.25 | 8.64 | 0.82 |
| BRT | 0.70 | 2.27 | 15.67 | 1.68 |

**Table S5.** Results of ten-fold cross-validation regression model of Artificial neural network (ANN), Support vector machine (SVM), Random Forest (RF), Boosted Regression Trees (BRT).

| Model | Model accuracy with Ten-cross-validation | | | |
| --- | --- | --- | --- | --- |
|  | R^2^ | RMSE  (t·ha^-1^·yr^-1^) | rRMSE  (%) | MAE  (t·ha^-1^·a^-1^) |
| ANN | 0.48±0.06 | 2.92±0.25 | 20.13±1.66 | 2.02±0.14 |
| SVM | 0.49±0.05 | 2.91±0.23 | 20.06±1.50 | 1.85±0.12 |
| RF | 0.52±0.04 | 1.15±0.01 | 19.42±1.52 | 1.89±0.12 |
| BRT | 0.52±0.05 | 2.82±0.25 | 19.42±1.54 | 1.96±0.14 |

**Table S6.** Hyper-parameters of four ML regression model of Artificial neural network (ANN), Support vector machine (SVM), Random Forest (RF), Boosted Regression Trees (BRT).

| Model | Hyper-parameters |
| --- | --- |
| ANN | Decay = 0.001, size = 19 |
| SVM | Kernel = radial, gamma=0.2, cost = 2.2 |
| RF | Mtry = 3 |
| BRT | Interaction depth = 10, n.trees = 150, shrinkage = 0.05 |

a

b

c


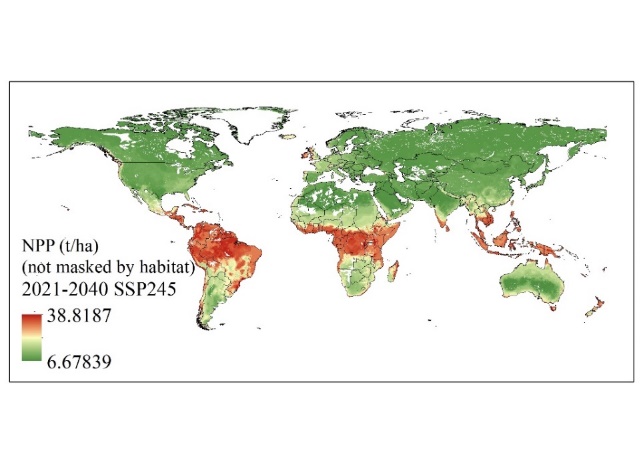

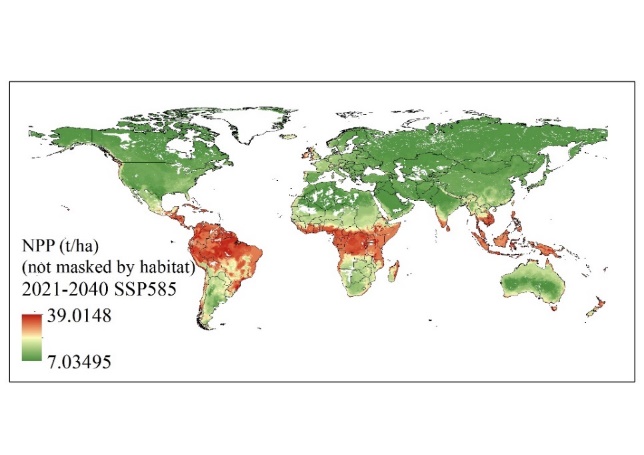

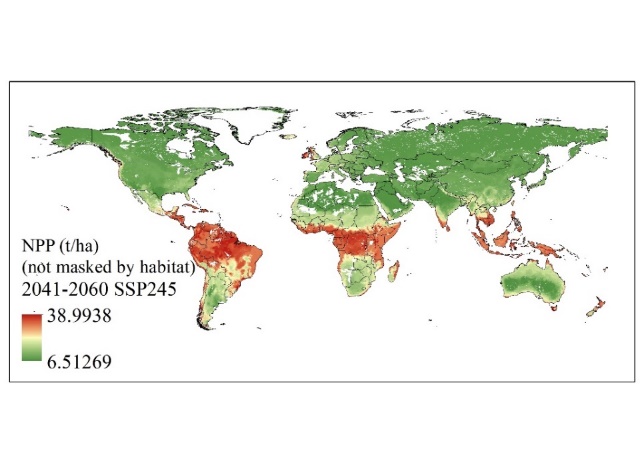

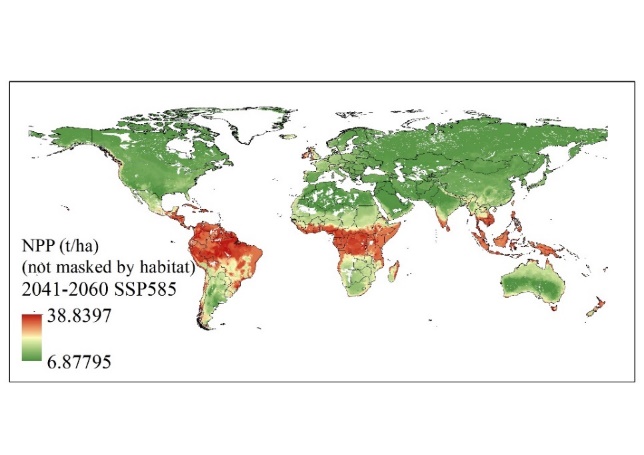

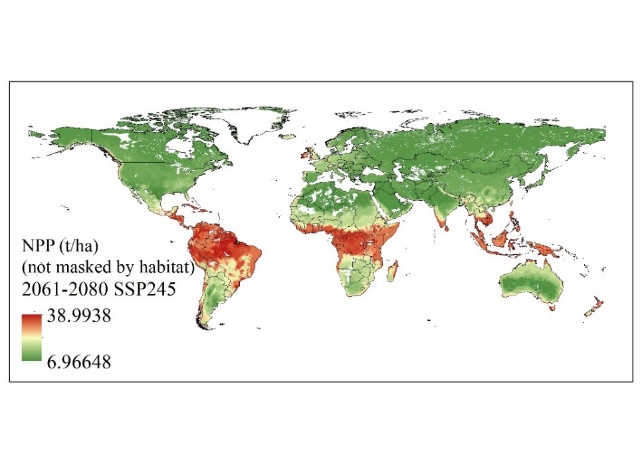

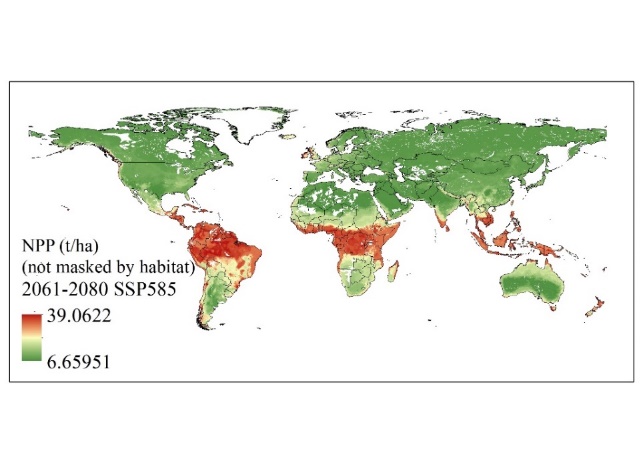

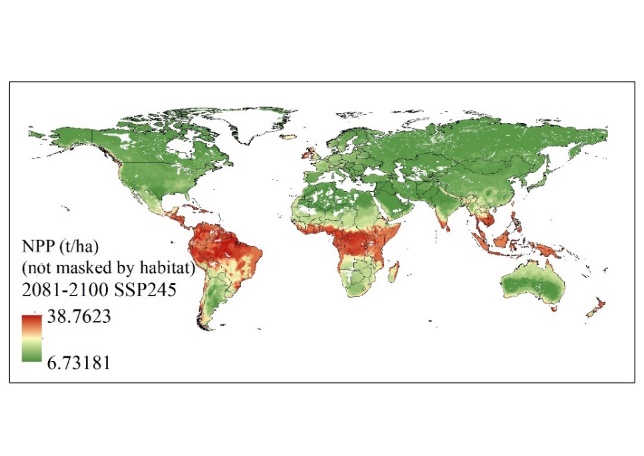

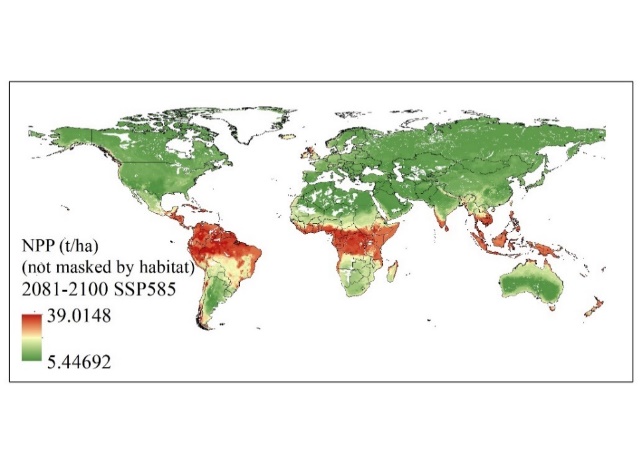

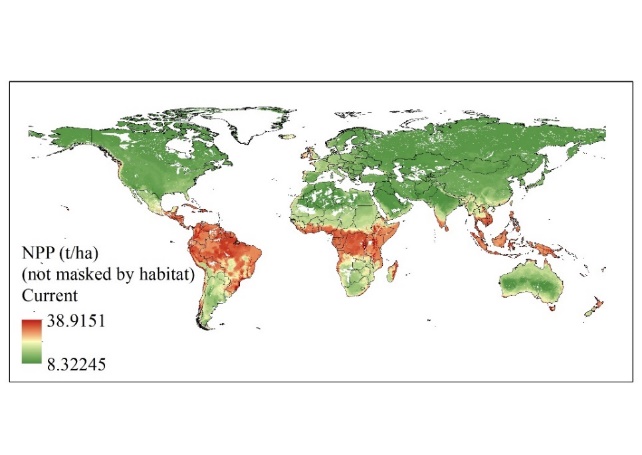


**Figure S1**. The global projected NPP map of Chinese fir plantation forests in the current and the future scenarios (not masked by suitable habitats).


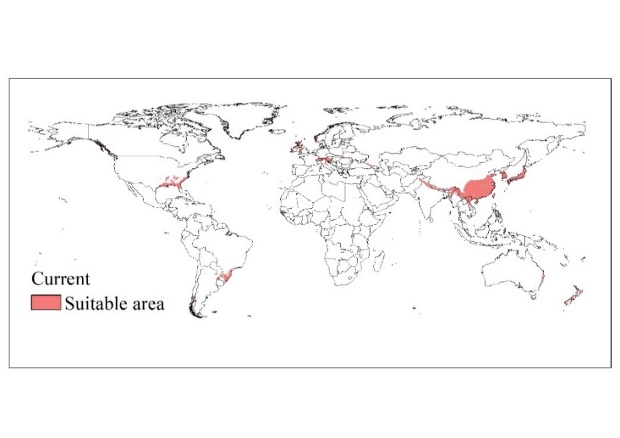


a

b

c


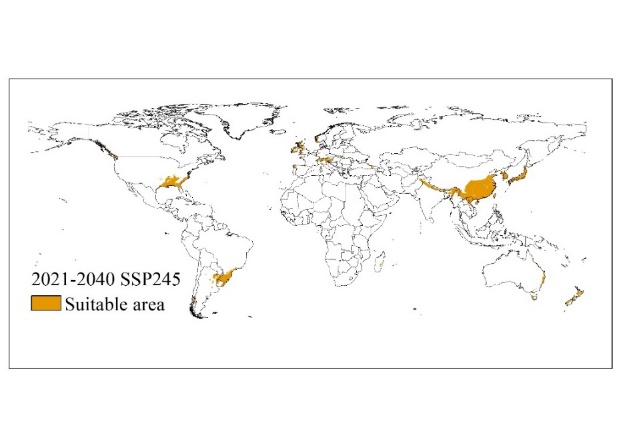

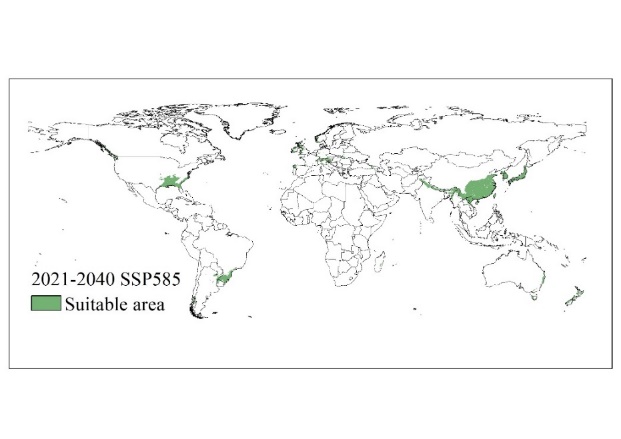

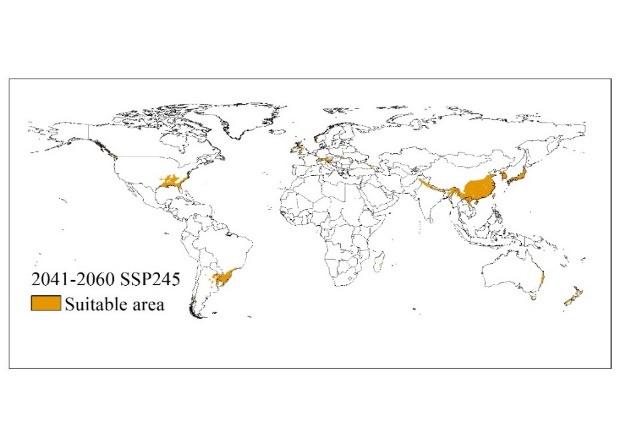

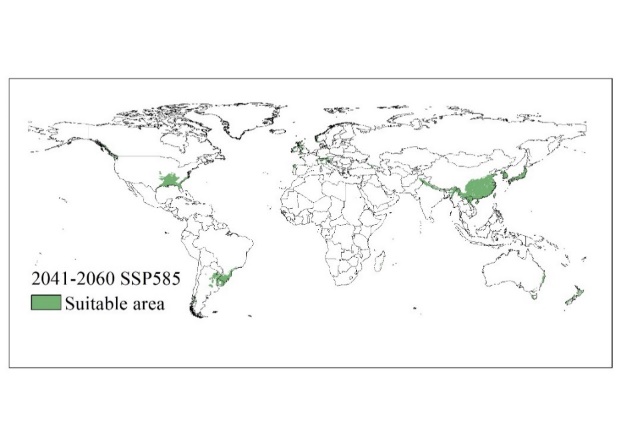

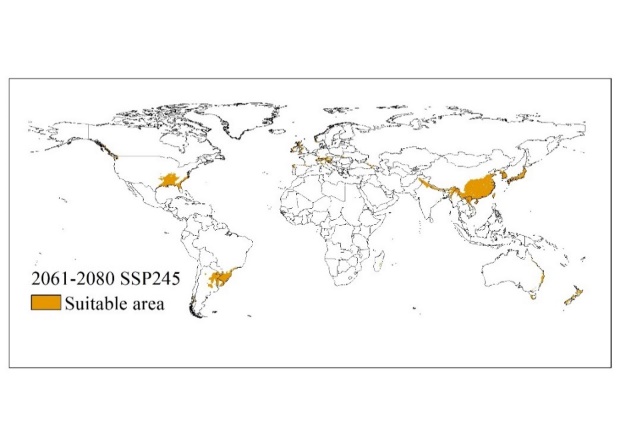

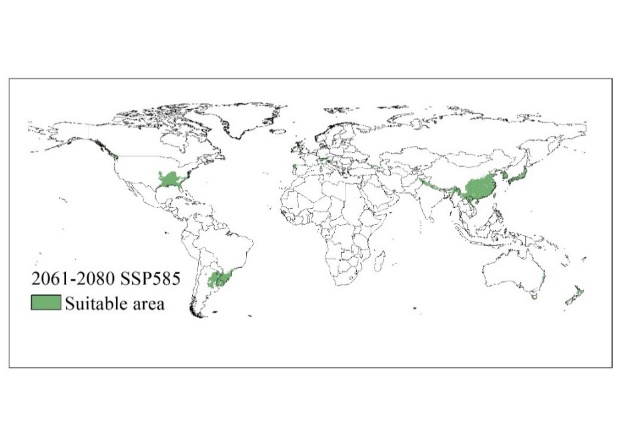

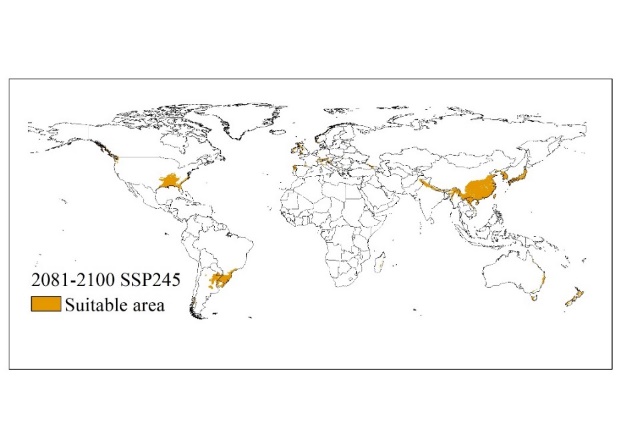

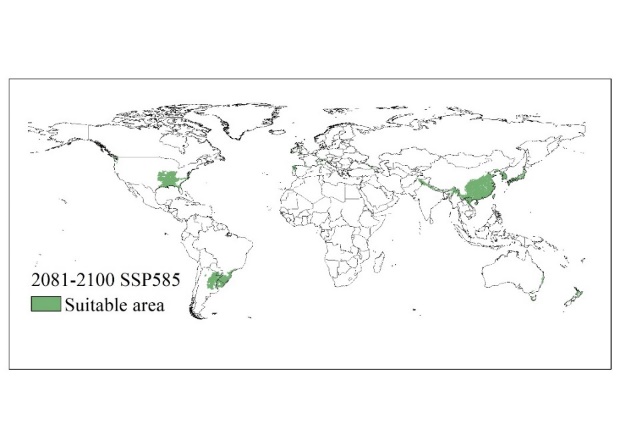


**Figure S2**. The global suitable areas of Chinese fir in the future scenarios.

a

b

c


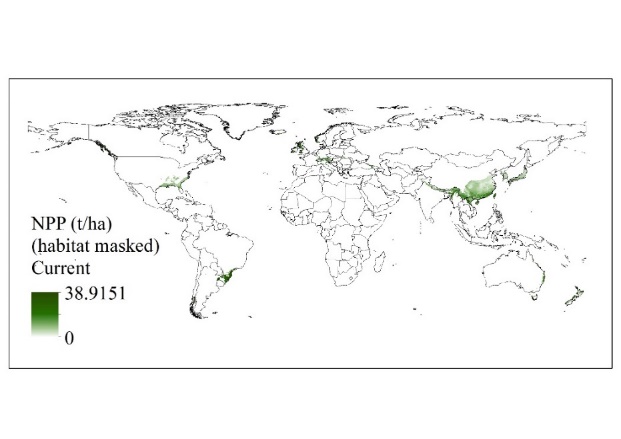

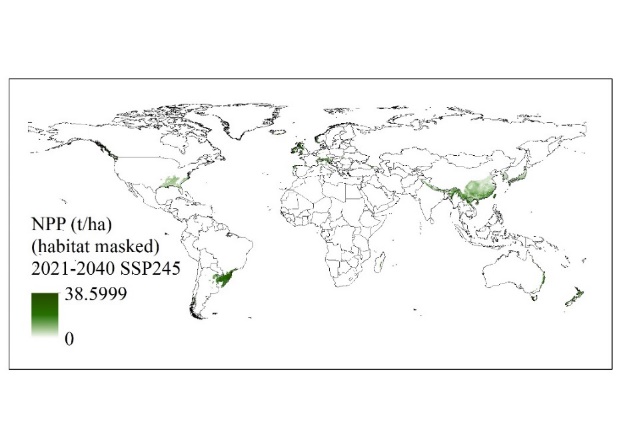

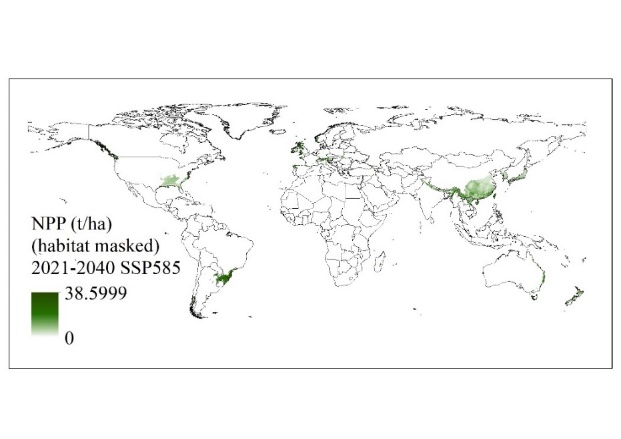

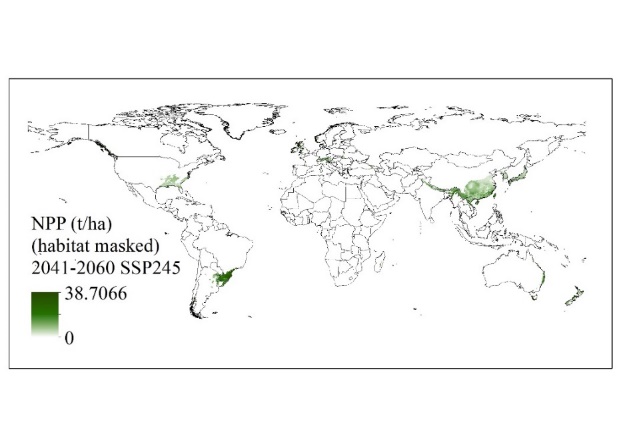

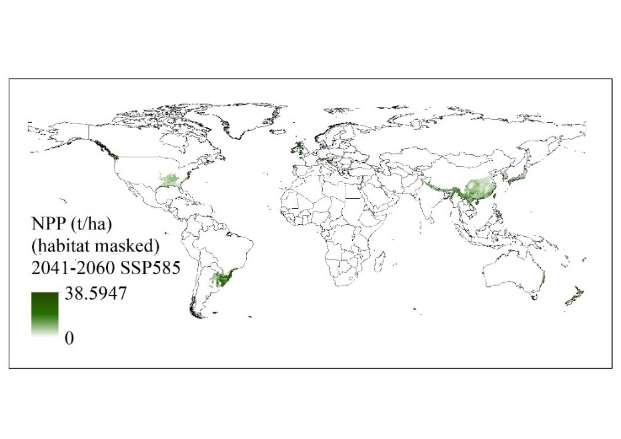

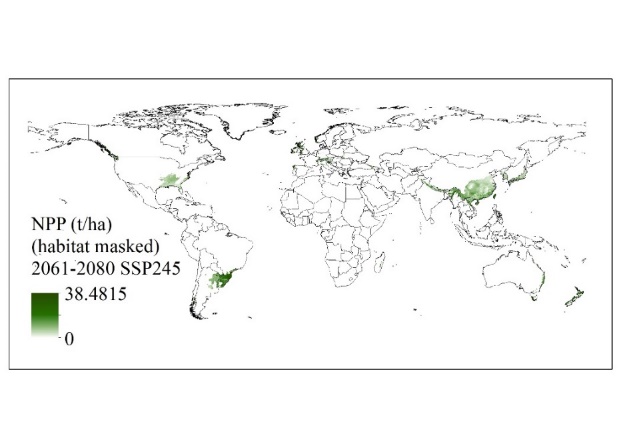

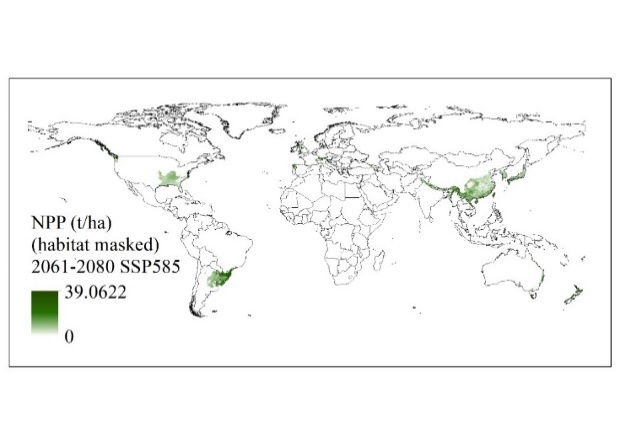

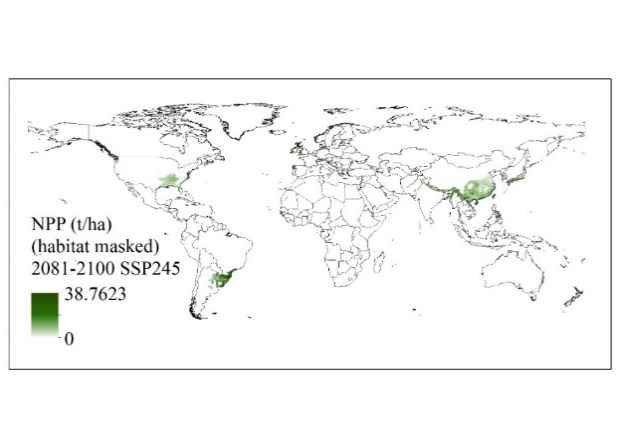

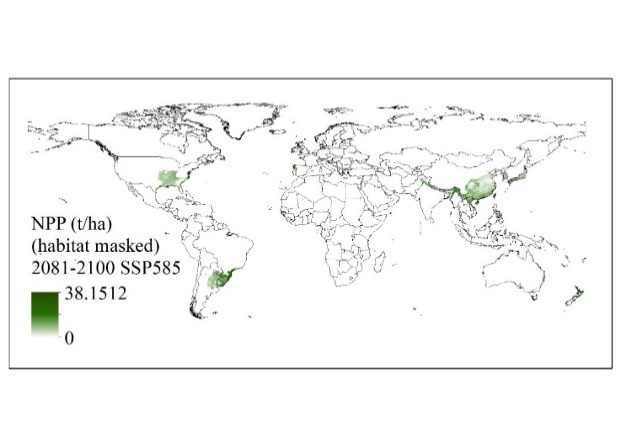


**Figure S3**. The global projected NPP of Chinese fir masked by suitable areas in the future scenarios.

b

c


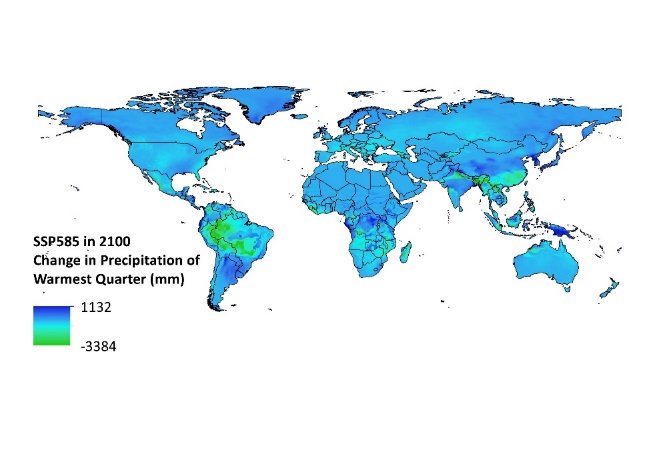

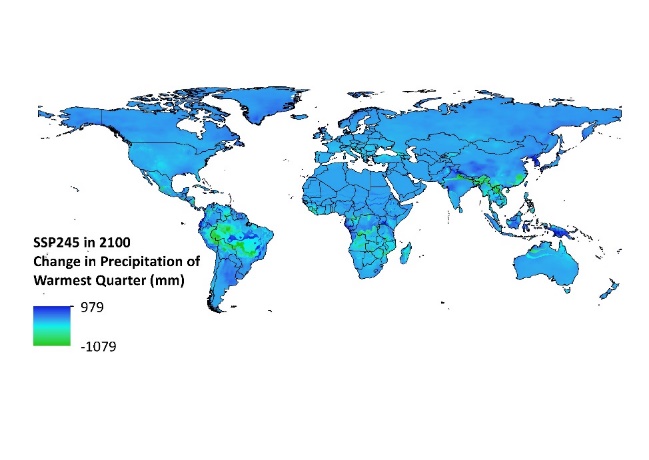

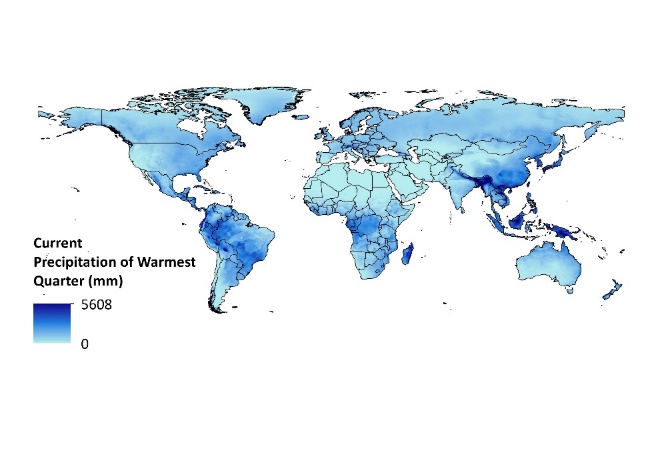

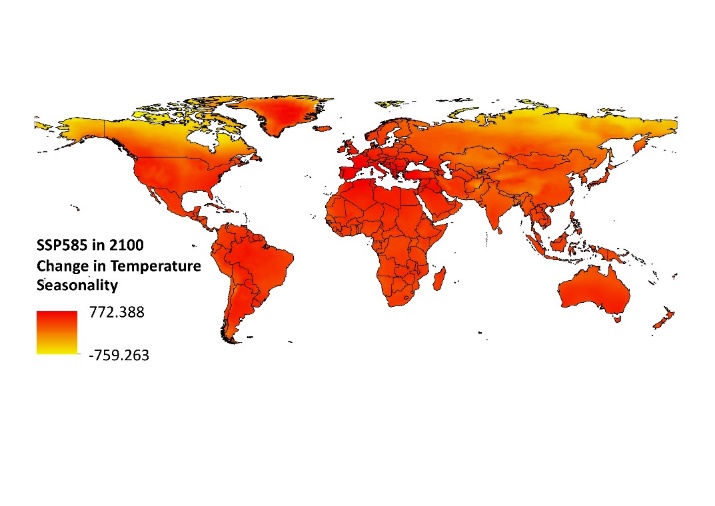

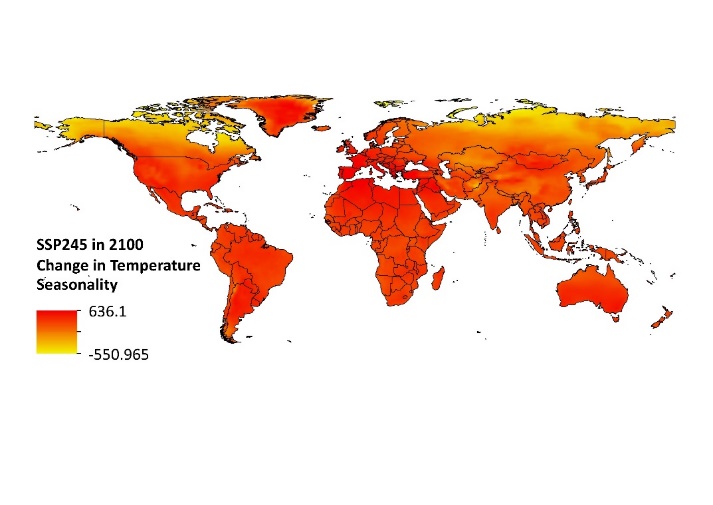

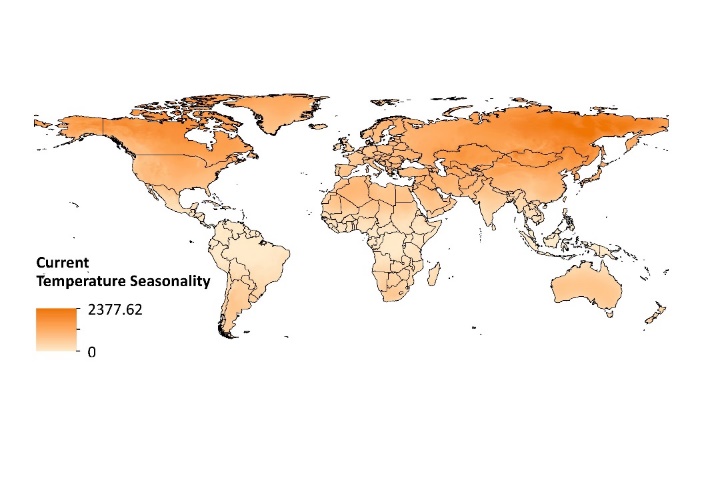

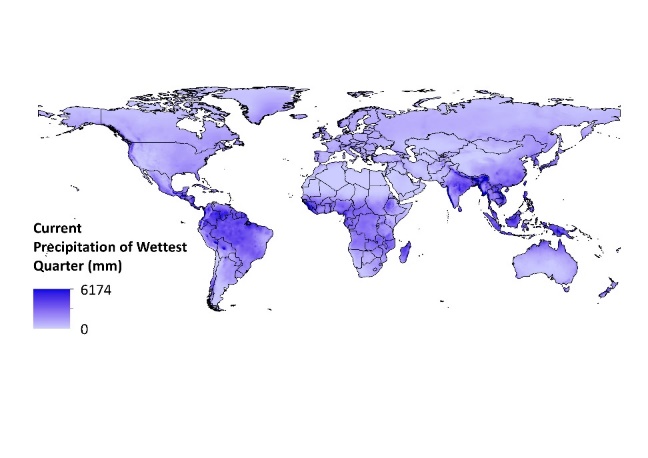

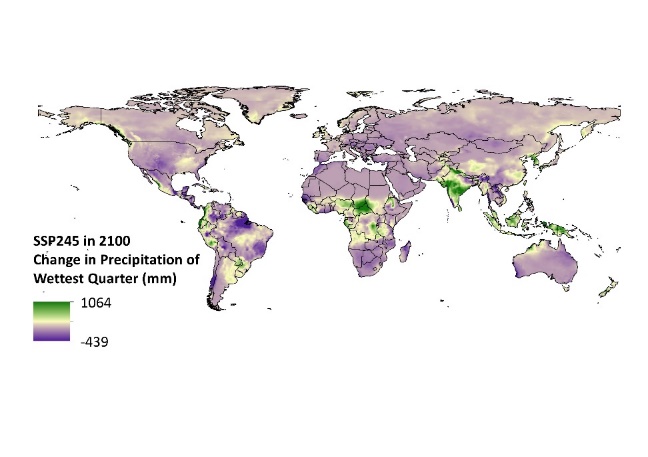

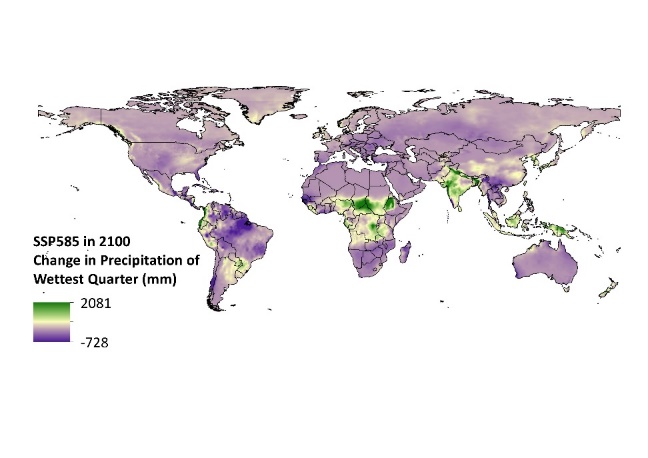


**Figure S4**. Major environmental factors of ENM of Chinese fir plantation forests in the current and their changes in the future scenario of SSP245 and SSP585 under 2100.


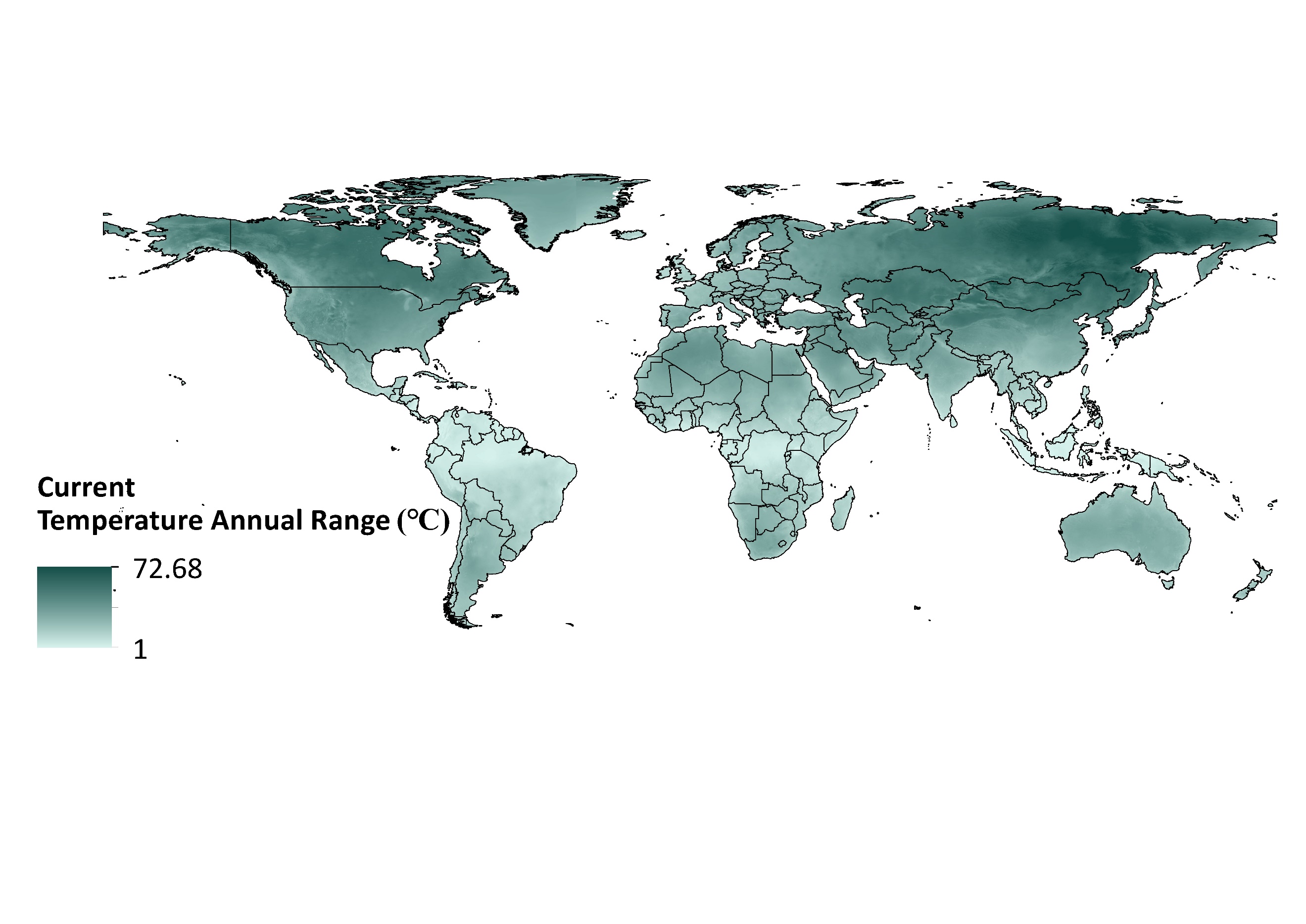

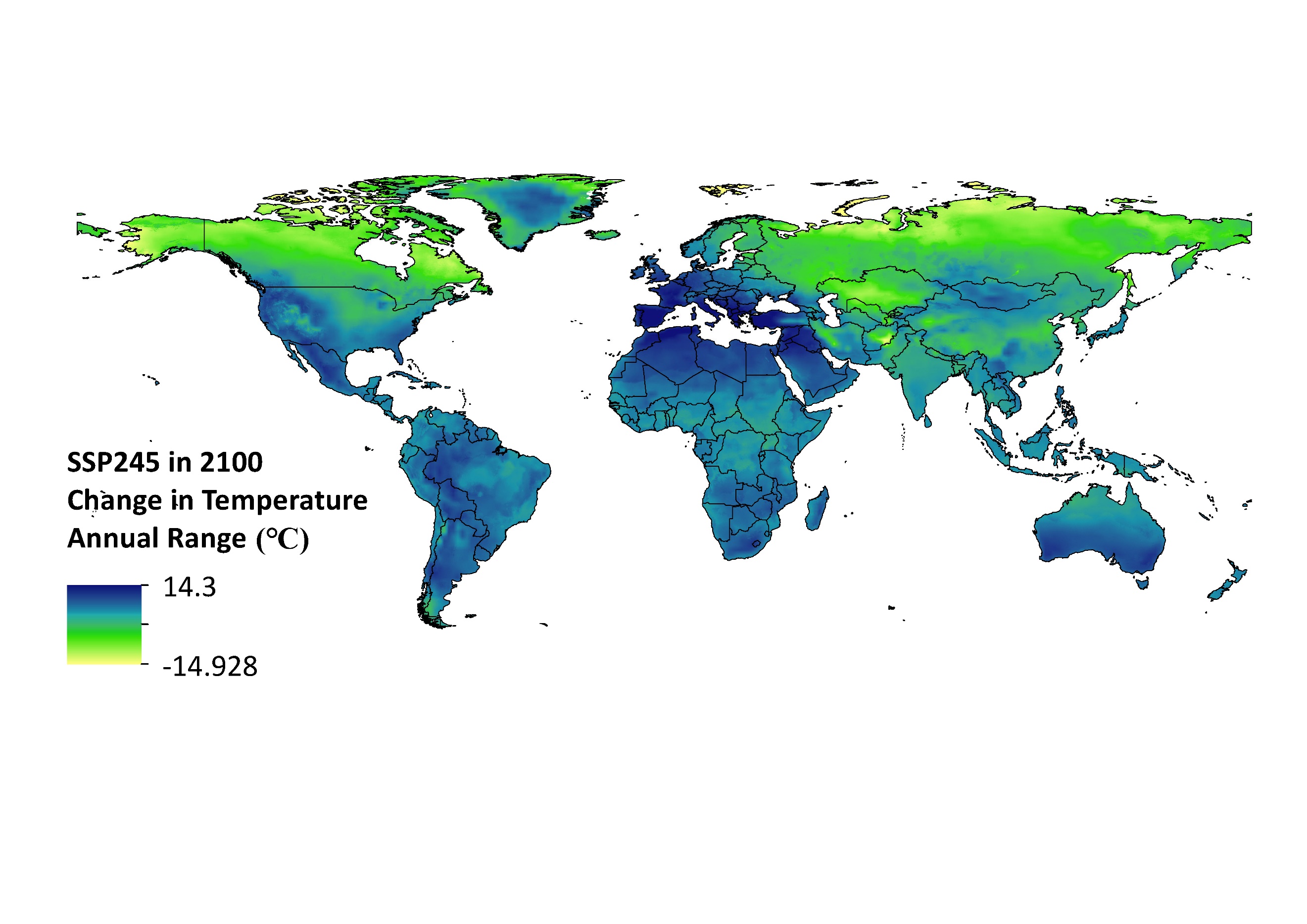

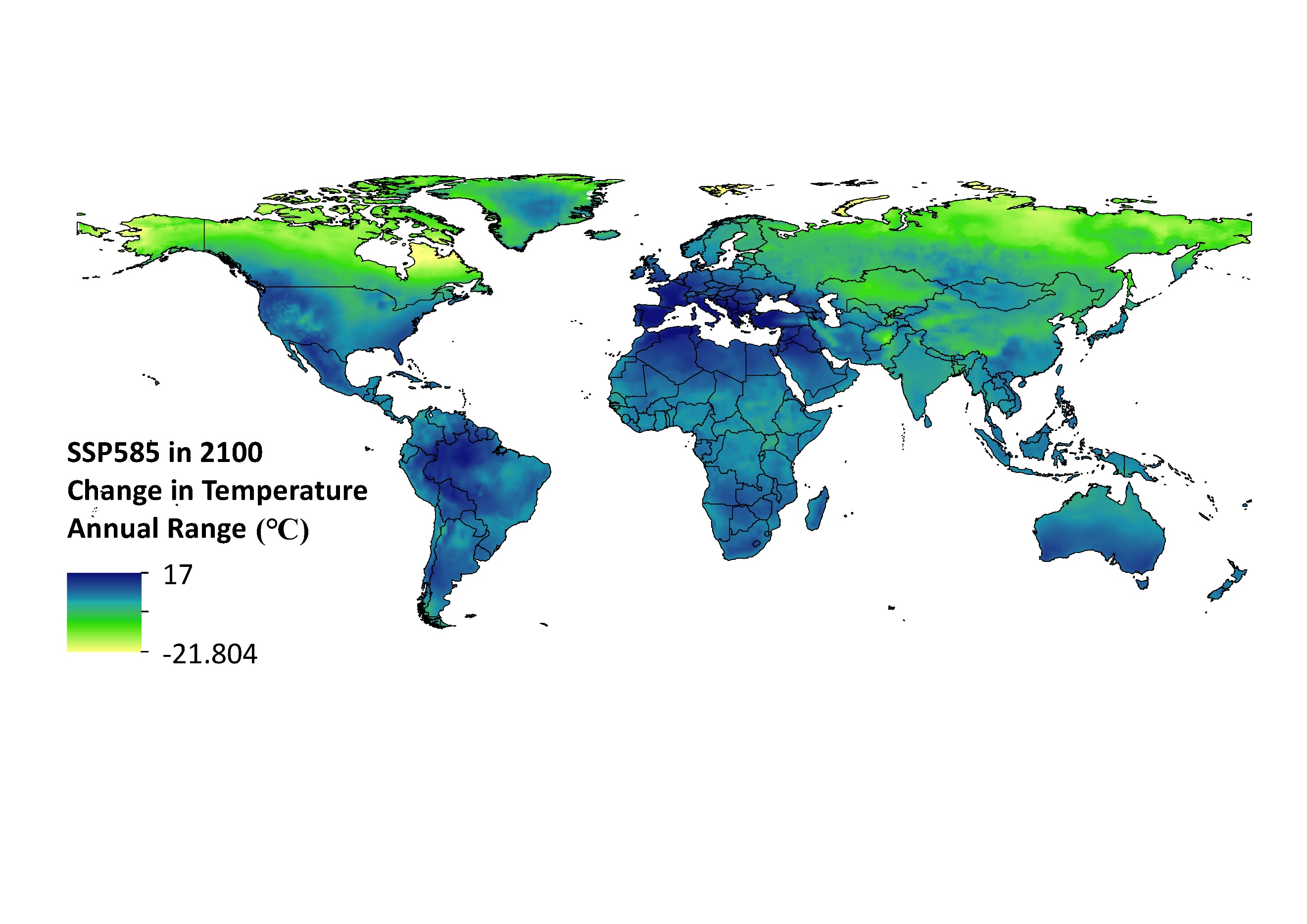

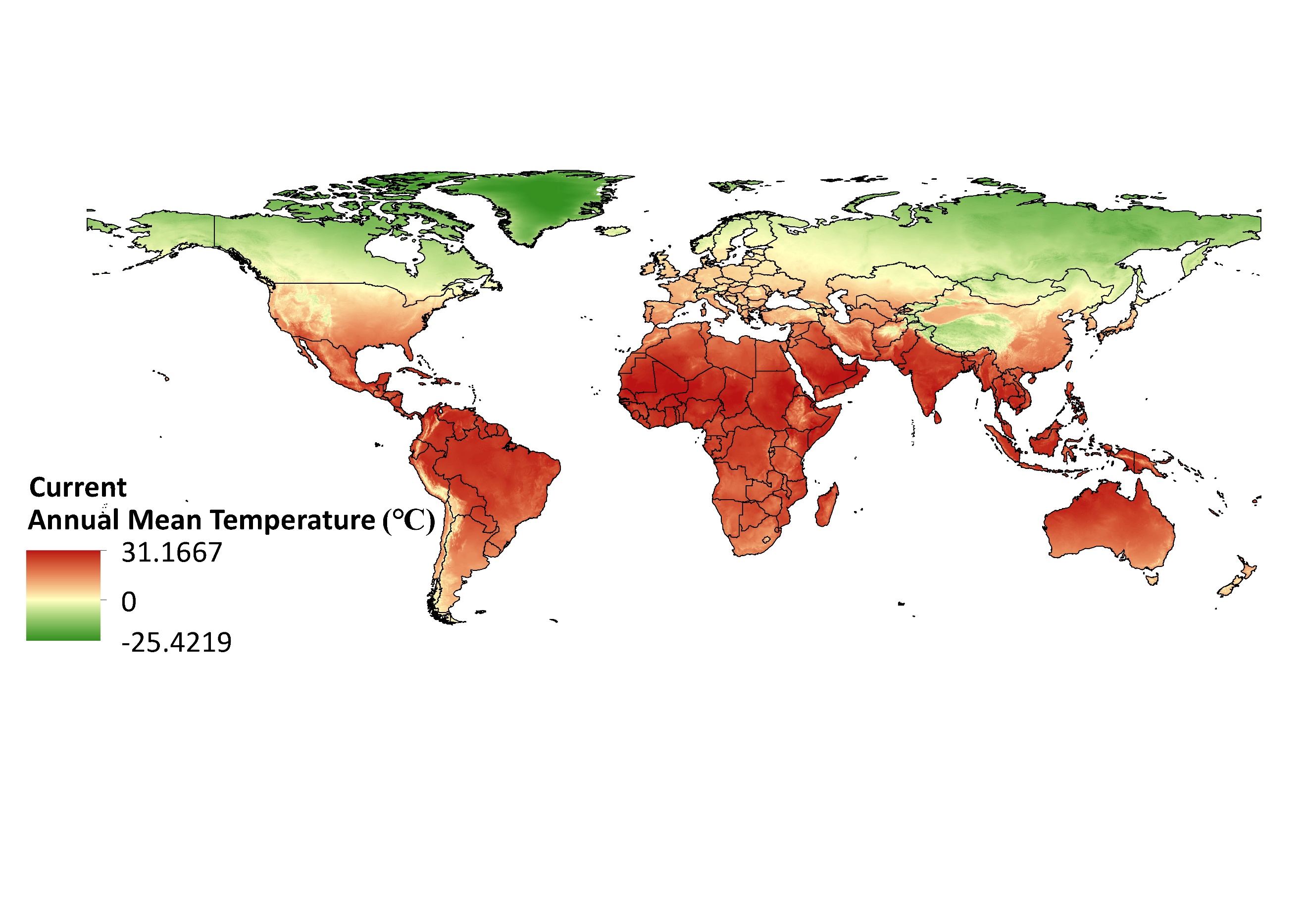

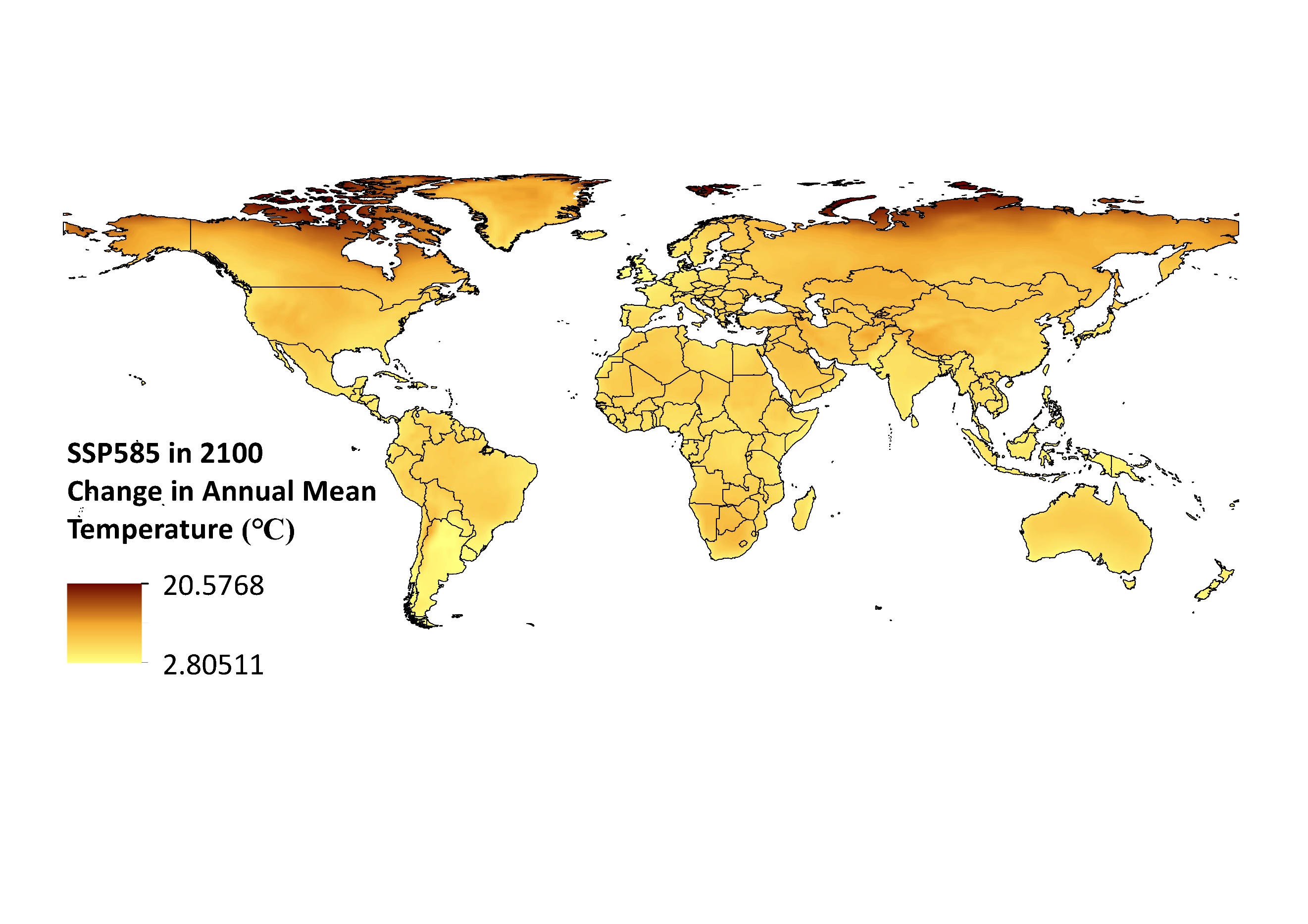

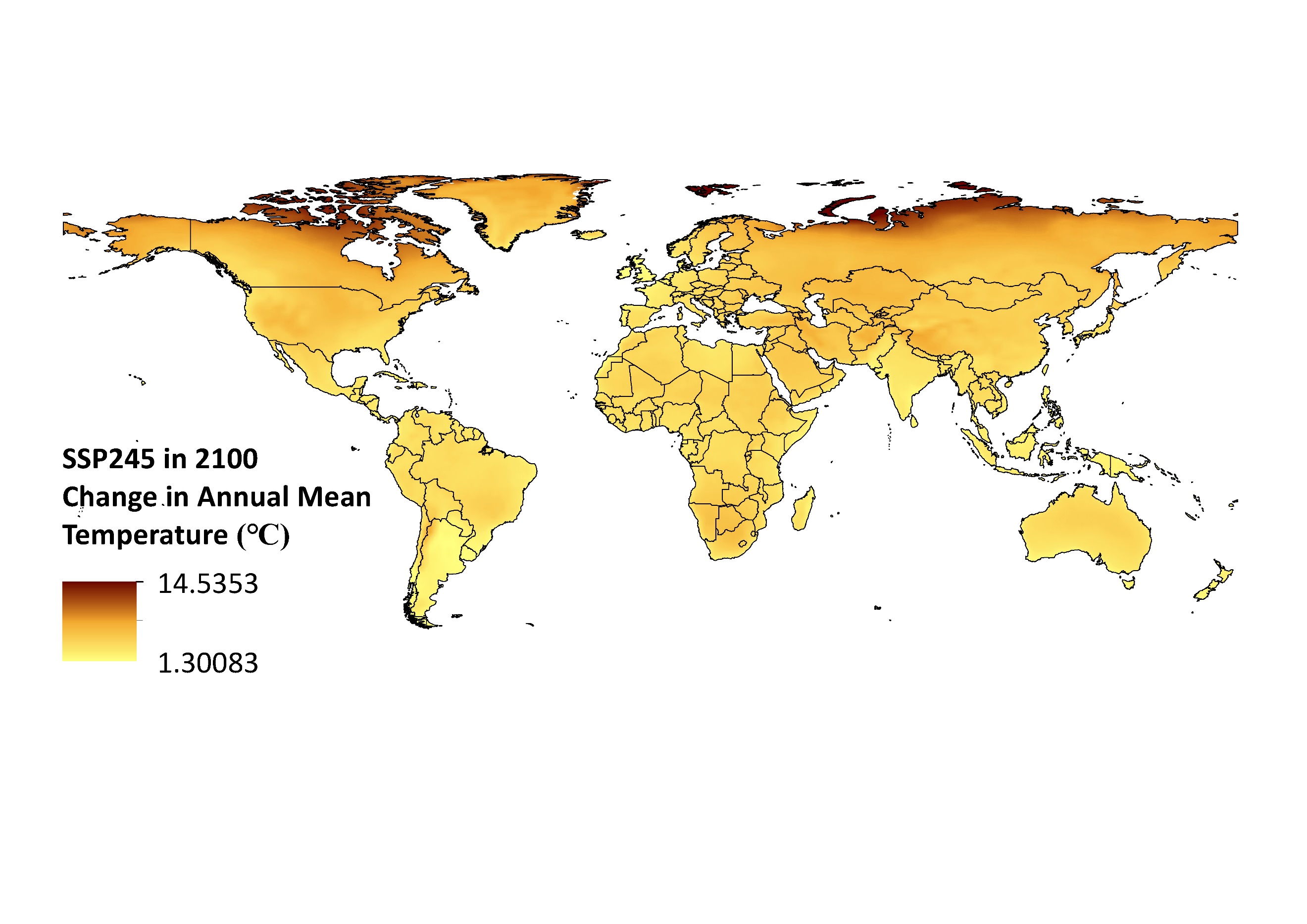

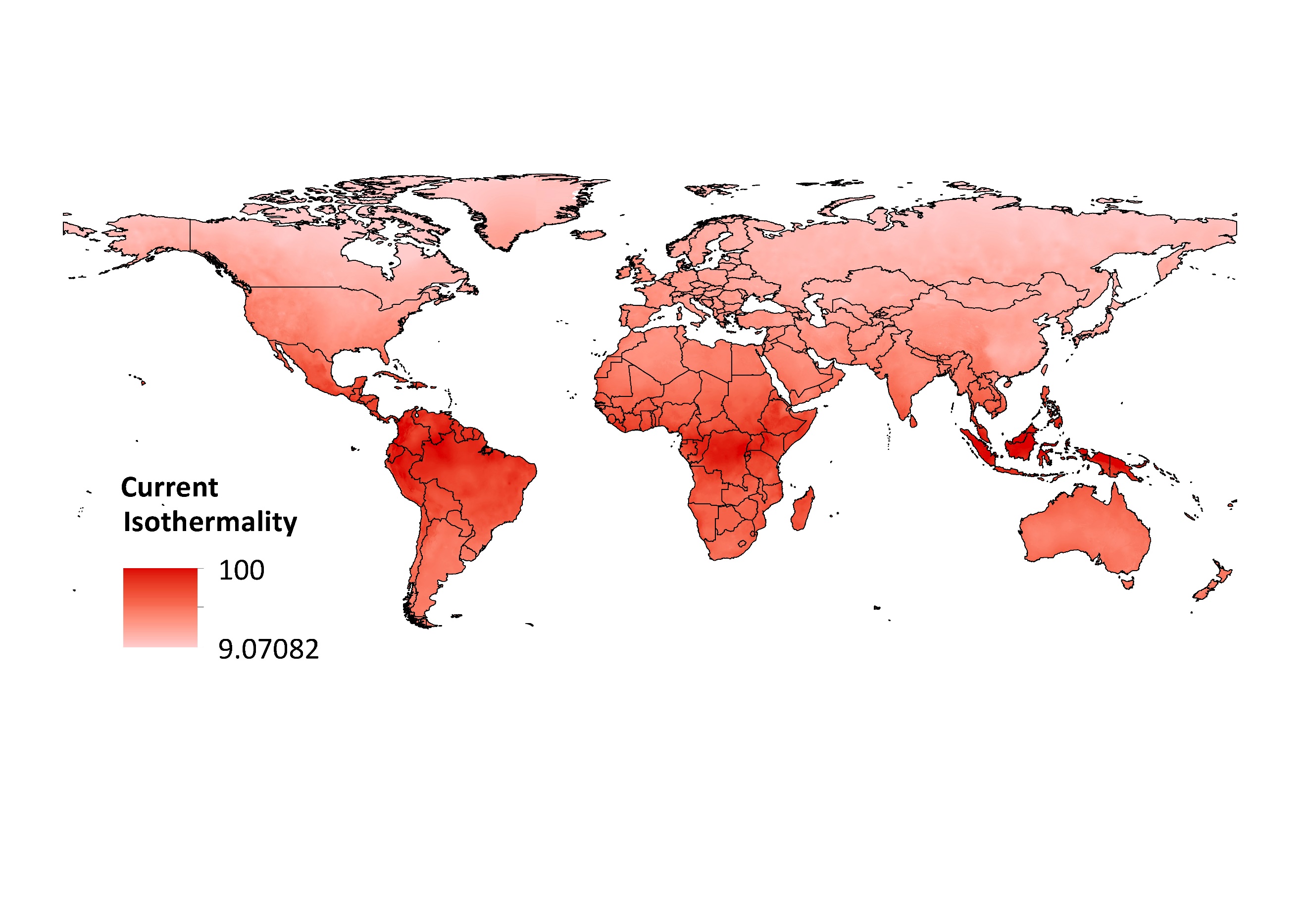

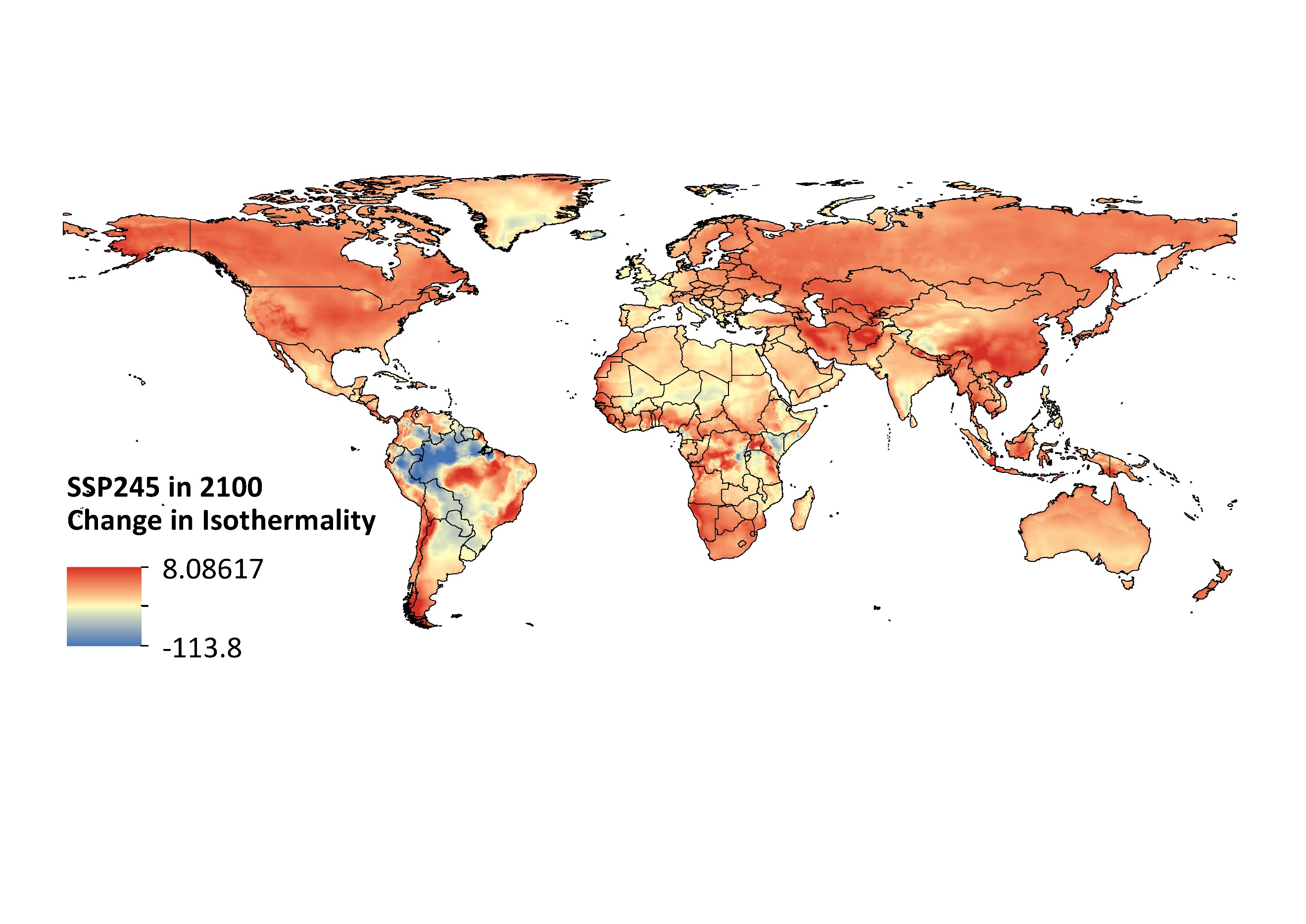

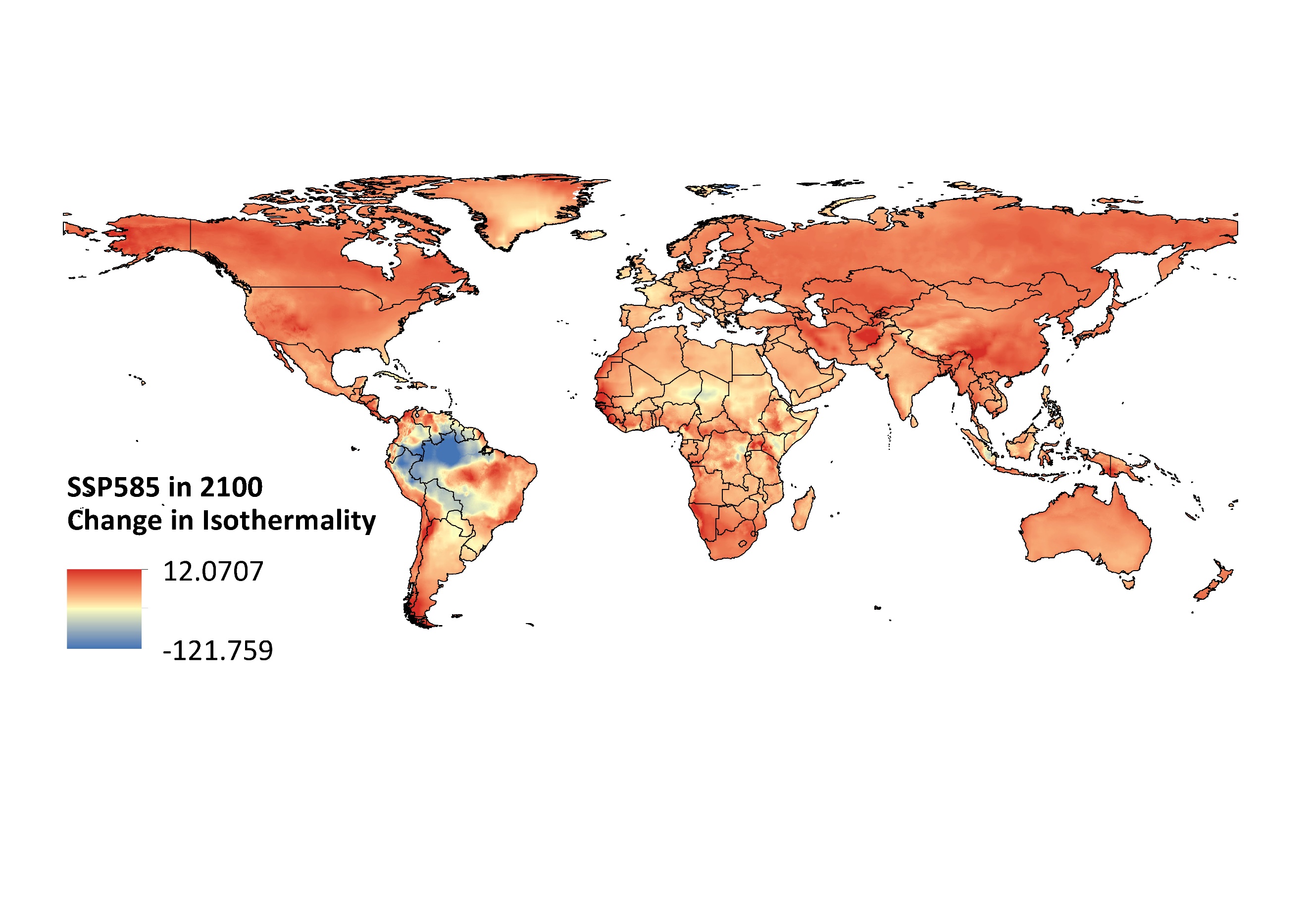


**Figure S5**. Major environmental factors of NPP regression model for Chinese fir plantation forests in the current and their changes in the future scenario of SSP245 and SSP585 under 2100.

**References**

1. S. Nandy *et al.*, Neural network-based modelling for forest biomass assessment. *Carbon Management* 8, 305-317 (2017).

2. B. Ripley, W. Venables, M. B. Ripley, Package ‘nnet’. *R package version* 7, 700 (2016).

3. Q. Xu, X. Lei, H. Zhang, A novel method for approaching the compatibility of tree biomass estimation by multi-task neural networks. *Forest Ecology and Management* 508, 120011 (2022).

4. D. Meyer *et al.*, Package ‘e1071’. *The R Journal* (2019).

5. L. Breiman, Random forests. *Machine learning* 45, 5-32 (2001).

6. A. Liaw, M. Wiener, Classification and regression by randomForest. *R news* 2, 18-22 (2002).

7. L. Breiman, A. Cutler, A. Liaw, M. Wiener, Package ‘randomForest’. *University of California, Berkeley: Berkeley, CA, USA* https://doi.org/10.1023/A:1010933404324 (2018).

8. J. Elith, J. R. Leathwick, T. Hastie, A working guide to boosted regression trees. *Journal of animal ecology* 77, 802-813 (2008).

9. G. Ridgeway, Generalized Boosted Models: A guide to the gbm package. *Update* 1, 2007 (2007).
